# A Lambda-evo (λ_evo_) phage platform for Zika virus E_DIII_ protein display

**DOI:** 10.1007/s00253-024-13380-3

**Published:** 2025-01-16

**Authors:** Honorio Negrete-Méndez, Guadalupe Valencia-Toxqui, Eva Martínez-Peñafiel, Oscar Medina-Contreras, Fernando Fernández-Ramírez, Edgar Morales-Ríos, Luis Janiel Navarro-González, Jesús M. Torres-Flores, Luis Kameyama

**Affiliations:** 1 Departamento de Genética y Biología Molecular, Centro de Investigación y de Estudios Avanzados del Instituto Politécnico Nacional, Av. Instituto Politécnico Nacional No, 2508, C.P. 07360 Mexico City, Mexico; 2 Department of Biology, Center for Phage Technology, Texas A&M University, College Station, TX USA; 3 Unidad de Investigación Epidemiológica en Endocrinología y Nutrición, Hospital Infantil de México Federico Gómez, Dr. Márquez No. 162, Mexico City, Mexico; 4 Unidad de Genética, Hospital General de México, Dr. Balmis 148, C.P. 06726 Mexico City, Mexico; 5 Departamento de Bioquímica, Centro de Investigación y de Estudios Avanzados del IPN, Instituto Politécnico Nacional No, 2508, C.P. 07360 Mexico City, Av Mexico; 6 Laboratorio Nacional de Vacunología y Virus Tropicales, Escuela Nacional de Ciencias Biológicas del IPN, Mexico City, Mexico

**Keywords:** Phage display, ZIKV, Lambda phage, Recombinant-protein, Directed evolution, λ_evo_, Lambda-evo phage

## Abstract

**Abstract:**

One of the most significant bacteriophage technologies is phage display, in which heterologous peptides are exhibited on the virion surface. This work describes the display of λ decorative protein D_λ_ linked to the E protein domain III of Zika virus (D_λ_-ZE_DIII_), to the GFP protein (D_λ_-GFP), or to different domain III epitopes of the E_ZIKV_ protein (D_λ_-TD), exhibited on the surface of an in vitro evolved lambda phage (λ_evo_). This phage harbors a gene *D* deletion and was subjected to directed evolution using *Escherichia coli* W3110/pD_λ_-ZE_DIII_ as background. After 20 days (20 cycles of dilution), the λ_evo_ phage developed a ~ 22% genome deletion affecting the non-essential λ *b* region, rendering a more stable phage that exhibited fusion proteins D_λ_-ZE_DIII_ or D_λ_-GFP but not D_λ_-TD. Despite the λ_evo_ system was able to decorate itself with the D_λ_-ZE_DIII_ protein, the production of viral particles was ~ 1000-fold lower than the λ wild-type, due to the unexpected D_λ_-ZE_DIII_ protein aggregation into bacterial inclusion bodies. Decorated phages (10^6^ PFU (plaque forming units)/100 µl) were inoculated into BALB/c mice, and subsequent dot blot and Western blot immunoassays proved the production of murine antibodies against ZIKV (Zika virus). This multipurpose λ_evo_ phage display platform may be used interchangeably with other more soluble peptides, providing better yields.

**Key points:**

• *λ*_*evo*_* platform for displaying recombinant peptides.*

• *Directed evolution to generate λ*_*evo*_* with more efficient decoration.*

• *Antigenic reaction in BALB/c mice by inoculating λ*_*evo*_* with recombinant peptides.*

**Supplementary Information:**

The online version contains supplementary material available at 10.1007/s00253-024-13380-3.

## Introduction

Bacteriophages are the most abundant entities in the biosphere (Mushegian 2020). When viruses infect their host, they can use the cell components to replicate their genome and synthesize their proteins to generate multiple virions and might also perform a series of additional functions. The interest in these ancient characteristics of viruses has resurged to produce novel biotechnological solutions (Mirzaei and Deng 2022). One of the most promising applications in biomedicine and basic research is the phage display, which consists in the exhibition of a desired foreign peptide fused to the phage protein that is exposed on the outer surface of its capsid (Jaroszewicz et al. 2021).

Several types of phages have been used for phage display (Hess and Jewell 2020; Jaroszewicz et al. 2021). For instance, filamentous phages M13 and fd of *Escherichia coli* K-12 were mainly used to construct peptide libraries in affinity assays. However, high-density displays were only achieved using short peptides (Jaroszewicz et al. 2021). Size, amino acid composition, and toxicity of the fusion protein remain as the main limitations of the M13 systems (Nicastro et al. 2014). On the other hand, tailed phages λ, T4, and T7 of *E. coli* K-12 represent more flexible platforms overcoming these limitations, allowing high-density display of large, hydrophilic or toxic peptides (Garufi et al. 2005; Nicastro et al. 2014; Hayes 2019). Thus, the selection of the phage system will depend on the type of peptides to be displayed.

Phage λ has been one of the most studied phages, with a comprehensive understanding of the proteins that constitute its capsid and tail, as well as its assembly process (Medina et al. 2010; Casjens and Hendrix 2015; Wang et al. 2022). It has an icosahedral capsid of ~ 60 nm in diameter mainly constituted by the E_λ_ and D_λ_ proteins and a tail of ~ 150 nm in length formed by the V_λ_ and other proteins (Lander et al. 2008; Nicastro et al. 2014; Wang et al. 2022, 2024). D_λ_ and V_λ_ have been used as fusion proteins for phage display (Jaroszewicz et al. 2021). Due to its localization, D_λ_ is an excellent candidate for this, as it binds to the external capsid surface as a protruding trimer, playing a vital role in the stabilization of the capsid during DNA packaging (Yang et al. 2000). Previous work has reported successful fusions to large proteins of up to 1000 amino acids, using either terminus of the D_λ_ protein (Mikawa et al. 1996; Pavoni et al. 2013), reaching high peptide display densities that could represent 90% of the ~ 415 copies of this protein per virion (Zucconi et al. 2001).

In vivo displays using the D_λ_ protein have been implemented under a two-gene system (wild-type and fused), where the wild-type *D* gene is usually encoded within the phage genome (Thomas et al. 2012) and the *D* gene fusion is expressed in cis or trans. This approach favors the stabilization of the decorated phages (Santini et al. 1998; Gamage et al. 2009; Hayes et al. 2010). However, the amount of fusion protein on the phage capsids could be reduced due to the higher affinity of wild-type D_λ_ for λ capsids (Nicastro et al. 2013). Therefore, this system can incorporate the D_am15_ variant instead of the wild-type D_λ_ (Thomas et al. 2012), to achieve a better control of the amount of D_λ_ produced (Mikawa et al. 1996; Nicastro et al. 2013). On the other hand, the phage λ D_λ_^‒^ platform allows in vitro decoration by adding purified soluble D_λ_ fusion proteins to phage preparations (Sternberg and Hoess 1995; Yang et al. 2000; Mattiacio et al. 2011).

The implementation of several λ-based platforms for the presentation of antigens or immunogens from different pathogens or cancer has been described. Gamage and Hayes reported a tandem display of porcine *Circovirus* 2 (PCV2) epitopes fused to the C-terminus of D_λ_ that could induce neutralizing antibodies against PCV2 (Gamage et al. 2009; Hayes et al. 2010). Simultaneous display of GFP and the transduction domain of the TAT_HIV_ protein using a λ carrier achieved antibody induction against GFP to a greater extent than a genetic immunization (Thomas et al. 2012). An in vitro approach using λ particles decorated with the human immunodeficiency virus Envelope protein (Env) purified from HEK 293-F cells also generated neutralizing antibodies. However, this antibody production could not overcome that obtained from conventional adjuvanted oligomeric protein (Mattiacio et al. 2011). The use of different HER2/neu-derived peptides (between 8 and 19 amino acids) for a breast cancer vaccine could induce an immune response in vitro and, importantly, could protect mice in a tumor challenge (Arab et al. 2018; Barati et al. 2018; Razazan et al. 2019). Finally, the cancer vaccine candidate SNS-301 against squamous cell carcinoma of the head and neck is currently in phase 1/2 clinical trials (Algazi et al. 2020).

In recent years, Zika virus (ZIKV) has emerged as a major global health problem due to its association with neurological disorders (e.g., Guillain-Barré syndrome, microcephaly) and no vaccine or treatment has been approved so far (Woodson and Morabito 2024). Due to its location on the external side of the viral membrane, the Envelope glycoprotein (E_ZIKV_) is the main target of antibodies, making it an important option for vaccine development (Dai et al. 2016). The domain III of E_ZIKV_ (ZE_DIII_) has demonstrated viability for the induction of several neutralizing and specific antibodies, which is the desired immune response in vaccine design for ZIKV (Wang et al. 2016; Robbiani et al. 2017; Shukla et al. 2020).

This work aimed to modify the phage λ through a physiological-genetic strategy to allow the formation of stable viral particles displaying ZE_DIII_. A directed in vitro evolution strategy was applied to obtain λ_evo_ phages, which were able to display such protein with a relatively low viral production rate but a high protein density. After the inoculation of these phages in a BALB/c mouse model, murine antibodies that recognized the E protein from the ZIKV were induced.

## Materials and methods

### Bacteria, plasmids, and bacteriophages

The bacterial strains, phages, and plasmids used in this study are listed in Supplemental Table S1. Strain W3110 was used for phage propagation, protein production, and infection assays. Strain DH5α was used for several purposes: plasmid constructs transformation, plasmid propagation, lysogen constructions, and mutagenesis by recombineering. The oligonucleotides used were synthesized by ADN ARTIFICIAL S. de R.L. de C.V., Irapuato, México, and are listed in Supplemental Table S2. Phage λ_cI857_ (harboring a thermosensitive CI repressor) was used to construct phages λΔD::KanR, λΔD, and λ_evo_.

### Media, enzymes, and bacterial growth conditions

Lysogenic broth (LB) culture medium and TMG (10 mM Tris–HCl pH = 7.8, 100 mM NaCl, 10 mM MgSO_4_, 0.1% gelatin), TN (10 mM Tris–HCl pH = 7.8, 100 mM NaCl), and TE (10 mM Tris–HCl pH = 7.8, 100 mM NaCl, 100 mM ethylene-diamine-tetra-acetic-acid (EDTA)) buffer solutions were used for phage dilution and were prepared according to (Sambrook and Russell 2001). Bacterial strains were grown in LB broth at 37 °C/200 revolutions per minute (rpm). The culture medium was supplemented with 100 μg/ml ampicillin (Amp), 50 μg/ml kanamycin (Kan), and 0.05 to 0.5 mM isopropyl β-D-1-thiogalactopyranoside (IPTG) final concentrations when required (all were purchased from Sigma-Aldrich, St. Louis, MO, USA). LB top agar was prepared with addition of 7 g/l bacteriological agar (BD, Franklin Lakes, NJ, USA). Phosphate-buffered saline (PBS) was used for Western blot and dot blot assays, and was prepared according to (Sambrook and Russell 2001). The restriction enzymes, T4 DNA ligase, and T4 PNK enzyme used in plasmid construction were obtained from New England Biolabs Inc. (Ipswich, MA, USA).

### Phage propagation and DNA extraction

The subsequent procedure was followed to obtain isolated phage plaques. From a λ_cI857_ phage stock (any other phage can be used), serial dilutions were prepared in TMG buffer (1:100), and 3 μl of each dilution were spotted on the bacterial lawn (4 ml of LB top-agar medium mixed with 400 μl of an overnight culture (O/N) of W3110). Once the tiny drops dried, the plates were incubated O/N at 37 °C. For phage propagation in liquid culture, 1 ml of an O/N culture of strain W3110 was mixed with 500 μl of a 1 M MgCl_2_/CaCl_2_ solution (1:1) and ten phage plaques. Phages were allowed to adsorb for 15 min at room temperature (RT), then 50 ml of liquid LB medium was added and incubated at 37 °C/200 rpm for approximately 5 to 6 h, until complete cell lysis was observed. Subsequently, 5 ml of chloroform was added, and the mixture was shaken for an additional 15 min to allow the complete release of phages. The lysate was transferred to a 50-ml conical tube and centrifuged at 6000 × *g* for 10 min to remove cell debris. The supernatant was decanted into a clean conical tube and stored at 4 °C. Finally, the lysate was titrated on a W3110 bacterial lawn. Phage DNA was extracted as described in Sambrook and Russell (2001).

### Construction of the mutant phages λΔD::KanR and λΔD

**Fig. 1:**
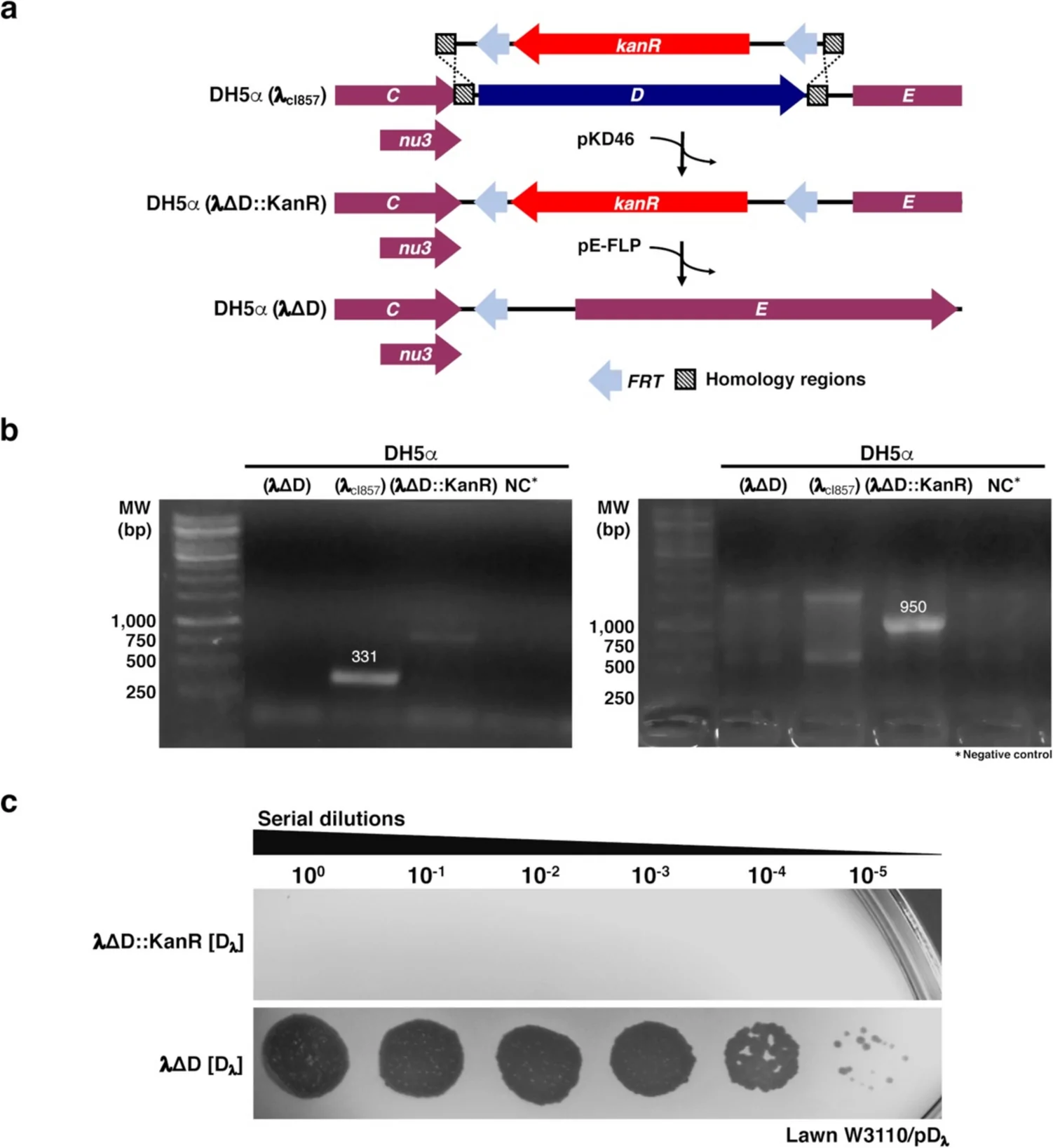
Construction of phage λΔD. **a** The λΔD lysogen was obtained by recombineering. Direct repeats of *FRT* flanking the KanR cassette are indicated as light-blue arrowheads, and the regions of homology upstream and downstream of the *D* gene are indicated as boxes with dashed lines. Neighboring genes *C*/*nu*3 (overlapped) and *E* are illustrated. The production of λ Red proteins from plasmid pKD46 promoted homologous recombination, resulting in the substitution of *D* gene by the KanR cassette. After obtaining the lysogen for λΔD::KanR, the kanamycin resistance cassette was removed by flippase FLP, which was produced from plasmid pE-FLP, resulting in phage λΔD. **b** PCR evaluation of the λΔD construct. Using the *D* gene primers (left gel), PCR amplification was only observed from λ_cI857_, producing an expected DNA fragment of 331 bp. With the kanamycin primers (right gel), PCR amplification was only observed for λΔD::KanR, yielding a DNA fragment of 950 bp. PCR amplification for λΔD was negative for both reactions. Template DNA used for each PCR corresponds to the distinct DH5α lysogens, as indicated. NTC, non-template control. **c** Production of viral particles from the engineered lysogens transformed with plasmid pD_λ_. The presence of lysis spots from serial phage dilutions were evaluated on a W3110/pD_λ_ lawn, and only the λΔD lysogen produced viral progeny

Construction of the λΔD::KanR mutant phage was performed by replacing the *D* gene in the DH5α(λ_cI857_) lysogen with the *kan* gene by recombineering (Fig. 1a), following the protocol of Datsenko and Wanner (2000) with slight modifications (Baba et al. 2006). The DNA fragment containing the kanamycin gene cassette (KanR) with flippase recombination target (*FRT*: gaagttcctatactttctagagaataggaacttc) repeats on each side and flanked by a 40-bp sequence upstream and downstream of the *D* gene was amplified by PCR using the primers D_Recomb-Fw and D_Recomb-Rv (Supplemental Table S2), and the genomic DNA of strain JW2787 as a template. The DNA fragment was cleaned with the GeneJet PCR purification kit (Thermo-Fisher, Waltham, MA, USA) and resuspended in nuclease-free water. Meanwhile, a DH5α(λ_cI857_) lysogen transformed with the pKD46 plasmid containing the *exo*, *bet*, and *gam* genes of phage λ was grown at 30 °C in 50 ml of LB medium with 100 μg/ml ampicillin and 1 mM L-arabinose, with shaking at ~ 180 rpm until reaching OD_600_ = 0.6. Then, the culture was concentrated 100-fold to make electrocompetent cells. The cells were washed three times with 10% ice-cold glycerol, and 20 μl of the treated cells and 300 ng of the purified PCR product were mixed and electroporated using a Cell-Porator 1600 device (Life Technologies GIBCO/BRL, Carlsbad, CA, USA) with the voltage amplifier, using the following settings: capacitance at 1180 μF, voltage at 220 V, and resistance at 16 kΩ, according to the manufacturer’s instructions. One milliliter of super optimal broth medium (SOB) was added to the shocked cells and left at room temperature O/N. Then, the mixture was spread on LB agar plates with 50 μg/ml kanamycin to select Kan-resistant transformant cells. Each candidate was analyzed by PCR, using specific primers: (1) For the KanR cassette and (2) for the *D* gene (Fig. 1; Supplemental Table S2).

The DH5α(λΔD) strain was further obtained from the DH5α(λΔD::KanR) lysogen. For this purpose, the lysogen was cured from the pKD46 plasmid, transformed with pE-FLP containing the FLP recombinase (Datsenko and Wanner 2000), and subsequently grown in 5 ml of LB-Amp (100 μg/ml) at 32 °C for 2 h. The bacteria were spread on LB and LB-Kan plates to assess the loss of the KanR cassette, and after several passages on plates, the strain was cured of the pE-FLP plasmid. Candidate colonies were evaluated by PCR to ensure the loss of the *D* and *kan* genes. Once confirmed, both strains were transformed with the pD_λ_ plasmid and cultured in 5 ml of LB-Amp at 32 °C for O/N. A 1/100 dilution was made in 5 ml of LB and cultured to an OD_600_ = 0.4 at 32 °C. Once the desired density was reached, the cultures were transferred to 42 °C for 15 min to induce prophages. Finally, the cultures were reheated at 32 °C for 3 h until total lysis was observed. The supernatants were titrated to assess the number of viral particles produced.

### ZIKV propagation, RNA extraction, and pJET-ZE_DIII_ plasmid construction

VERO cells (ATCC, Manassas, VA, USA) were seeded in F75 flasks and grown to 80% confluence using M199 medium (supplemented with carbonate and 6% FBS). Infection was performed by adding 500 μl of a Zika viral stock (~ 1 × 10^6^ plaque forming units (PFU)/ml) (GenBank: KY631494.1; Supplemental Table S1) and allowing it to adsorb at 37 °C in a humidified incubator supplemented with 5% CO_2_ with gentle shaking every 10 min. The culture was incubated for 5–6 days until lysis was observed, and the supernatant was clarified by centrifugation at 4000 × *g* for 30 min. Viral RNA extraction was performed using the QIAMP viral RNA extraction kit (Qiagen, Venlo, Netherlands), then it was aliquoted and stored at − 80 °C until its use. cDNA was obtained by reverse transcription of viral RNA using the iScript Select cDNA Synthesis kit (BioRad, Hercules, CA, USA) with random primers, following the manufacturer’s protocol.

The sequence for domain DIII of the E_ZIKV_ gene was amplified from total cDNA using specific primers (E_ZIKV_(DIII)-Fw and E_ZIKV_-Rv). Restriction enzyme cleavage site (*Hin*dIII) and a 6 × histidine sequence were incorporated at the 3′ end of the ZE_DIII_ primer. It is noteworthy that in the 3′ region, a TKA codon was inserted immediately after the sequence encoding the 6 histidines (6xHis), resulting on either a stop (TAA) or serine (TCA) codon. For this work, only the clones containing the TCA codon were selected. The amplification product contained the *Xba*I site on one end and the *Hin*dIII and *Sac*I sites on the other end, and then it was cloned into the transition vector pJET1.2/blunt to obtain plasmid pJET-ZE_DIII_−6xHis.

### Construction of pD_λ_ and pD_λ_−6xHis plasmids

Plasmids pD_λ_ and pD_λ_−6xHis were constructed by amplifying the *D* gene from phage λ using different sets of oligonucleotides. For pD_λ_, the oligos D_STOP-SD-Fw and D_Stop-Rv were used. For pD_λ_−6xHis, the oligonucleotides D-Fw and D_HisTag-Rv were used, in which the reverse primer containing a sequence encoding for 6xHis was incorporated downstream the 3′ end of *D* gene. The amplification products were cloned into the transition vector pJET1.2/blunt to generate pJET-D_λ_ and pJET-D_λ_−6xHis. The fragments were re-amplified with oligos pJET1.2-Fw and pJET1.2-Rv provided by the manufacturer, which anneal at both flanks of the vector cloning site, adding 50 bp to each end of the insert. This new amplicon was digested with *Eco*RI and *Hin*dIII restriction enzymes and the released fragment was purified using the GeneJet PCR purification kit (ThermoFisher, Waltham, MA, USA). The fragment was then ligated into the pKQV4 expression vector previously digested with the corresponding restriction enzymes.

### Construction of the pD_λ_-ZE_DIII_-L-6xHis plasmid

The D_λ_-ZE_DIII_-L-6xHis fusion was constructed by 2-step overlapping extension PCR. The first fragment (F1) was amplified from pJET-D_**λ**_ using the D_Link-Fw and Link(*Xho*I)-Rv oligonucleotides, where the reverse oligo removes the *D* gene stop codon and inserts 48 bp at its 3′ end that generate the overlapping sequence. The second fragment (F2) was amplified from pJET-ZE_DIII_−6xHis using the Link(*Xho*I)-Fw and E_ZIKV_-Rv oligonucleotides, in which the forward primer inserts 45 bp at the 5′ end as an overlapping sequence. Both fragments were purified with GeneJet PCR purification kit (ThermoFisher, Waltham, MA, USA) and quantified. An intermediate product was generated using 10 ng of each fragment, which were mixed in a PCR reaction without oligonucleotides and were amplified for 34 cycles using an annealing temperature of 55 °C. Finally, 1 μl of the intermediate amplicon was taken and re-amplified using D-Fw and 2Step-Rv oligos, to obtain the final fusion product containing the D_λ_ and ZE_DIII_ coding fragments with a 72 bp linker in between (D_λ_-ZE_DIII_-L-6xHis; Supplemental Fig. S1a); this fusion fragment was inserted into the pJET1.2/blunt transition vector (generating the vector pJET-D_λ_-ZE_DIII_-L-6xHis) and then subcloned into the *Eco*RI/*Hin*dIII sites of the pKQV4 expression plasmid to generate pD_λ_-ZE_DIII_-L-6xHis. After plasmid sequencing, three nonsynonymous mutations were found (A634G, A710G, and A733G). The first two mutations were repaired with the specified nucleotide using the QuickChange-II Lightning kit (Agilent Technologies, Santa Clara, CA, USA); the third mutation was not modified.

### Construction of the pD_λ_-ZE_DIII_-6xHis plasmid

Plasmid pD_λ_-ZE_DIII_−6xHis was constructed from plasmid pD_λ_-ZE_DIII_-L-6xHis, where the linker region between D_λ_-ZE_DIII_-L-6xHis was replaced by cutting the *Pst*I and *Xba*I restriction sites. For this replacement, a pair of complementary oligonucleotides (LinkFlex-1 and LinkFlex-2) was designed to generate a 7 amino acid linker to evaluate the effect of a shorter linker in the display of the fusion protein (Supplemental Fig. S1b). Oligonucleotides were phosphorylated by mixing 1X of T4 ligase buffer, 100 μM of each oligo, 10 units of T4 PNK enzyme and bringing the reaction to a final volume of 50 μl on ice. The reaction was incubated at 37 °C for 30 min and then at 65 °C for 20 min. Subsequently, 50 mM NaCl was added and the pair of oligonucleotides were annealed and incubated at 95 °C for 5 min, allowing them to cool slowly to room temperature for 15 min. Finally, the annealed fragment was inserted into the plasmid pD_λ_-ZE_DIII_-L-6xHis using the *Pst*I and *Xba*I sites.

### Construction of the pD_λ_-ZE_DIII_

The plasmid pD_λ_-ZE_DIII_ was constructed based on the pD_λ_-ZE_DIII_−6xHis plasmid, by site-directed mutagenesis to completely remove the 6xHisTag region and insert a stop codon in its place, using oligonucleotides (Xtag-Fw and Xtag-Rv) and the QuickChange-II Lightning kit (Agilent Technologies, Santa Clara, CA, USA), following the manufacturer’s instructions.

### Construction of the pD_λ_-TD-6xHis, pD_λ_-GFP, and pD_λ_-GFP-L plasmids

For pD_λ_-TD-6xHis plasmid construction, three linear epitopes within the sequence of the E_ZIKV_ protein were selected, and their nucleotide sequences were optimized for expression in *E. coli.* We designed in silico a fusion DNA fragment containing the nucleotides for these three epitopes and the corresponding nucleotide sequences for the GGSG linkers between them. In addition, we added a 6xHis coding sequence at the 3′-terminal and the *Xba*I and *Hind*III restriction nucleotide sites on its flanks. This entire fusion (see Supplemental Fig. S1d) was synthesized by ADN ARTIFICIAL S. de R.L. de C.V. and obtained as a plasmid. The fragment was cut and ligated into the pD_λ_-ZE_DIII_−6xHis plasmid using the aforementioned restriction endonucleases, removing the portion of the ZE_DIII_ sequence from the plasmid (Supplemental Fig. S1c).

pD_λ_-GFP and pD_λ_-GFP-L plasmids were constructed by amplifying the GFP protein gene with oligonucleotides (GFP-Fw and GFP-Rv), then cloning the resulting product into the pJET1.2/blunt vector, and subcloning into the *Xba*I and *Hin*dIII sites of the pD_λ_-ZE_DIII_−6xHis and pD_λ_-ZE_DIII_-L-6xHis plasmids, respectively, removing the last portion of the ZE_DIII_ sequence.

### Obtention of the λ_evo_ phage by directed evolution

First, a primary liquid lysate of phage λΔD was prepared in the W3110/pD_λ_ strain, in which phage λΔD stabilized with D_λ_ protein produced in trans (λΔD [D_λ_]) was obtained. The display of fusion proteins in phage λ lacking the D_λ_ protein usually results in unstable phages or very low viral titer (Zanghi et al. 2005; Nicastro et al. 2013; Supplemental Fig. S2). In order to enhance the stability and decoration obtained in λΔD, a directed evolution approach (Meyer et al. 2012) was performed by mixing 10 μl of a λΔD [D_λ_] liquid lysate, 5 μl of 1 M MgCl_2_, and 15 μl of a W3110/pD_λ_-ZE_DIII_−6xHis O/N culture. Phages were allowed to adsorb for 15 min at 37 °C, then 5 ml of LB supplemented with 3 mM MgCl_2_ were added and incubated at 37 °C/180 rpm O/N. The next day, 1 ml of the liquid phage lysate was treated with 100 μl of chloroform, and this was used to generate a subsequent phage lysate with freshly grown W3110/pD_λ_-ZE_DIII_−6xHis bacteria. Every 5 days, a sample was tested by infection assays on a W3110/pD_λ_ lawn until the phage titer persisted the same for 2 weeks (20 cycles of dilution).

### Sequencing and annotation of λ_evo_ phage genome

The λ_evo_ phage was sequenced using a NovaSeq 6000 sequencer (Illumina, San Diego, CA, USA), through the services provided by the SeqCenter (Pittsburgh, PA, USA). The raw sequence data was deposited in NCBI Sequence Read Archive database (SRA) under the BioProject accession number PRJNA1168660. The complete genome was assembled using the Galaxy interface (usegalaxy.eu). The quality of the raw reads was evaluated using FastQC version 0.12.1. (http://www.bioinformatics.babraham.ac.uk/projects/fastqc/); low-quality reads were removed with Trimmomatic version 0.38.1 (http://www.usadellab.org/cms/?page=trimmomatic), and the reads were assembled into a single contig using Shovill-Spades version 1.1.0 (https://github.com/tseemann/shovill) with K-mer set as default. This contig was annotated using the Genome Annotation Service in BV-BRC with PHANOTATE (McNair et al. 2019), setting the λ genome as a reference. To evaluate the genomic differences between λ_evo_ and λ_wt_ (RefSeq: NC_001616.1) genomes, a comparative genome analysis was performed using the ViPTree (https://www.genome.jp/viptree/) server.

### Evaluation of fusion protein display on λΔD and λ_evo_ platforms

The W3110 strain was transformed with plasmids expressing different gene fusions (Supplemental Fig. S1a and S1b) for phage display. Liquid lysates of λΔD or λ_evo_ were prepared in a final volume of 5 ml with addition of 3 mM MgCl_2_. The lysates were centrifuged for 1 min at 21,000 × *g* to eliminate cellular debris. Serial dilutions were prepared with TN buffer to evaluate decorated stable phages, and TN buffer supplemented with 10 mM MgCl_2_ to evaluate phage viability; the dilutions were spotted on W3110 and W3110/pD_λ_ lawns.

### D_λ_-ZE_DIII_-6xHis protein purification under denaturing conditions for in vitro decoration

The D_λ_-ZE_DIII_−6xHis protein was purified using Ni–NTA agarose, following the Qiagen specification guide with slight modifications. W3110/pD_λ_-ZE_DIII_−6xHis bacterial cultures were grown at 37 °C in LB broth with 100 μg/ml ampicillin O/N. The next day, cultures were diluted 1:100 with LB broth and grown up to a density of OD_600nm_ = 0.3; at this point, cultures were induced with 0.5 mM IPTG for 3 h. After the induction, cells were centrifuged and harvested at 4200 × *g* for 10 min.

For purification under denaturing conditions, the cell pellet was suspended in lysis buffer (8 M urea, pH = 8.0) containing 10 mM phenyl-methyl-sulfonyl-fluoride (PMSF; a serine protease inhibitor) and 2% EDTA-free SIGMAFAST™ protease inhibitor cocktail (Sigma-Aldrich, St. Louis, MO, USA). Samples were left in a rotatory shaker O/N at room temperature. Samples were sonicated using the Soniprep-150 (MSE, Cholet, France) device at 10 micron amplitude with five cycles of 30 s on and 30 s off pulses; the samples were left for an additional two hours in a rotatory shaker. The lysate was clarified by centrifugation at 24,000 × *g* for 30 min. Ni–NTA agarose was applied to the clarified cell lysate and the mixture was incubated with rotatory shaking for 12 h at 4 °C. Afterwards, it was washed three times with a washing buffer (8 M urea pH = 6.4), and proteins were eluted four times using elution buffer (8 M urea pH = 4.5).

The purified proteins were placed in dialysis tubes with Spectra MWCO 7 kDa membranes (Spectrum labs, Irving, TX, USA) and dialyzed with a 10 mM Tris–HCl pH = 7.2, 100 mM NaCl and 10% glycerol (J.T.Baker, Phillipsburg, NJ, USA) buffer. Four changes of dialysis buffer, with a time of 8 h between each change, were performed to reconstitute the protein, which was functional as observed in subsequent in vitro decoration assays. The concentration of purified D_λ_-ZE_DIII_−6xHis protein was determined by spectrophotometry using the Bradford method (Kielkopf et al. 2020). These dialyzed proteins were stored at − 70 °C until further use.

### Anti SI-D_λ_-ZE_DIII_-6xHis polyclonal antibody production

Reconstituted protein (40 μg/50 μl) mixed with TiterMax Classic (TiterMax, Norcross, GA, USA) 1:1 was inoculated by subcutaneous injection at the base of the tail of 6-week-old BALB/c mice (*n* = 4) (Harlan, Mexico City, Mexico), using a 1-ml insulin syringe (BD, Franklin Lakes, NJ, USA). Prior to the inoculation, blood was drawn to assess pre-immunity. At 24 days post-inoculation, a second blood sample was obtained to verify the immune response. Blood samples were incubated for 1 h at 37 °C and 1 h at 4 °C. Samples were then centrifuged at 7000 × *g* for 15 min at 4 °C, and the sera were tested in Western blot experiments (Sambrook and Russell 2001) to assess their ability to detect purified D_λ_-ZE_DIII_−6xHis protein. This super-immune serum (anti-SI-D_λ_-ZE_DIII_−6xHis) was subsequently used to evaluate the fusion protein display on phage particles throughout several steps of the work by dot blot immunoassays (Mishra 2022; Gamage et al. 2009). Samples dropped on a nitrocellulose membrane were stained with Ponceau S (Sander et al. 2019) to evaluate the total protein from the different samples.

### In vitro phage-capsid decoration

Phages λ_evo_ and λ_evo_ [D_λ_-ZE_DIII_−6xHis] were decorated in vitro with the refolded recombinant protein D_λ_-ZE_DIII_−6xHis. Fifty microliters of liquid lysates were incubated with different concentrations of refolded protein (1, 2, 10, 20, 30, and 40 ng/μl) with rotatory shaking O/N at 4 °C. Serial dilutions were prepared with TN buffer and spotted on a W3110/pD_λ_ lawns to determine whether the purified D_λ_-ZE_DIII_−6xHis protein stabilized the decorated phages by increasing their viral titer.

### Stability assay without cations

The effect of EDTA on the stability of λ and λ_evo_ phages was evaluated. One hundred microliters of each phage lysate (~ 10^8^ PFU/ml) were incubated at 37 °C for 15 min in TE buffer (100 mM EDTA) (1:10). To assess the remaining phage viability, serial dilutions of the treated phages were subsequently prepared with TN buffer and spotted on a W3110/pD_λ_ lawn.

### Phage sample preparation for transmission electron microscopy (TEM)

Ten microliters of dialyzed CsCl-purified bacteriophages (1 × 10^8^ PFU/ml) were deposited on a Formvar carbon-coated grid (Electron Microscopy Science, Hatfield, PA, USA) and incubated for 1 min. The excess liquid was removed with filter paper, and then, 10 µl of 1% uranyl acetate (Electron Microscopy Science, Hatfield, PA, USA) was applied for 30 s and removed. Samples were analyzed using the electron transmission microscopes JEM-1400 (JEOL, Akishima, Japan) at 100 kV.

### Phage inoculum preparation for immunization

The phage lysates were incubated with DNAse (1 μg/ml) and RNAse (1 μg/ml) for 30 min at 37 °C and then were treated with chloroform and centrifuged at 4600 × *g* for 30 min. The supernatants were recovered and filtered with 0.45-μm membranes (Millipore, Burlington, MA, USA) to eliminate bacterial debris.

Phages were precipitated with 16% polyethylene glycol 8000 (PEG-8000) and 1.4 M NaCl, and then CsCl purification was performed. To eliminate endotoxins, we followed the protocol of Aida and Pabst (1990). The lysates were incubated with 2% Triton X-114 for 30 min at 4 °C with shaking, followed by incubation at 37 °C for 10 min to ensure detergent separation and centrifugation at 21,000 × *g* for 10 min at room temperature. The supernatant was recovered, and this process was repeated twice, evaluating the viral titer at each time. Several samples underwent in vitro decoration, and all samples were dialyzed with TN buffer.

### Evaluation of the immune response against D_λ_-ZE_DIII_-6xHis displayed by λ_evo_

An experimental design using groups of 6-week-old BALB/c mice (*n* = 3 per group) (Harlan, Mexico City, Mexico) received subcutaneous inoculation at the base of the tail with (a) 100 μl of phage solution containing 10⁶ PFU, (b) 100 μl of phage solution containing 10⁷ PFU, or (c) 100 μl of purified recombinant protein solution (1 μg). The inoculation protocol entailed three administrations: an initial inoculation followed by two booster inoculations at 2 and 4 weeks post-initial inoculation. Blood samples were collected from all mice 6 weeks after the first inoculation. To isolate serum for immunological analysis, pre-immunization and post-immunization blood samples were subjected to a two-step incubation process at 4 °C for 1 h followed by 37 °C for 1 h. Subsequently, samples were centrifuged at 7000 × *g* for 15 min at 4 °C. The supernatants constituting the serum fraction were pooled for each of the immunization groups and were used for further analysis.

### Immune sera evaluation

The gene coding the soluble ectodomain of E_ZIKV_ protein (residues 1 to 409) (GenBank accession number: WJY01991.1) was inserted into an expression vector to generate pRSET-ZIKV/E_S_ (Supplemental Table S1), which was a generous gift from Dr. Dan I. Zavala-Vargas. The viral protein was produced in the *E. coli* SoluBL21 strain, forming inclusion bodies (IB). Induced cells were lysed by sonication, and the IB were then denatured for 16 h in buffer containing 20 mM Tris–HCl [pH = 9.0], 500 mM NaCl, 1 mM dithiothreitol (DTT) and 8 M urea, shaking at room temperature. Refolding of denatured protein E was performed using the dropwise method described by Dai et al. (2016) with some modifications. In summary, denatured protein was added drop by drop into the refolding buffer (20 mM Tris–HCl [pH = 7.5], 100 mM NaCl, 100 mM L-arginine, 1 mM PMSF, 0.4 mM oxidized glutathione, 0.2 mM reduced glutathione, and 5% [v/v] glycerol) with continuous shaking at 4 °C overnight. The refolded protein was then concentrated by centrifugation (1650 × *g* at 4 °C) using an Amicon Ultra 15 (Merck Millipore, Burlington, MA, USA) concentrator with a 30 kDa cutoff membrane. Purified protein was stored in a buffer containing 20 mM Tris–HCl [pH = 8.0], 50 mM NaCl and 5% (v/v) glycerol.

E_ZIKV_ and Gp17_021_−6xHis (Valencia-Toxqui et al. 2023) purified proteins, as well as CsCl purified λ_evo_ [D_λ_] samples were dropped on a nitrocellulose membrane to perform dot blot analysis (Mishra 2022; Gamage et al. 2009) using as primary antibodies the different immune sera pools previously obtained. Same samples were resolved by SDS-PAGE and transferred to a nitrocellulose membrane to confirm the recognition of λ_evo_ [D_λ_-ZE_DIII_] serum by Western blot (Sambrook and Russell 2001).

## Results

### Construction of λ phage with D gene deletion (λΔD)

Phage λΔD was constructed through a 2-step recombination process using a DH5α(λ_*c*I857_) lysogen (see “Materials and methods” section for details). Briefly, the first step consisted in the replacement of the *D* gene with a KanR cassette (Fig. 1a). The lysogen DH5α(λ*c*I_857_) expressing the genes *exo*, *bet*, and *gam* was transformed with the KanR cassette (a previous PCR product obtained from the strain JW2787; Supplemental Table S1), which resulted in the generation of DH5α(λΔD::KanR); the pKD46 plasmid was subsequently cured. In the second step, the KanR cassette was removed by flippase-mediated recombination using the *FRT* sequences flanking it, thus generating the DH5α(λΔD) lysogen (Fig. 1a); the pE-FLP plasmid was cured afterwards. The recombinant lysogens were evaluated by PCR, with primers flanking the *D* and the *kan* genes (Fig. 1b; Supplemental Table S2). The absence of *D* and *kan* genes in the final DH5α(λΔD) construct was validated. As expected, the DH5α(λ_*c*I857_) lysogen generated a 333 bp DNA fragment corresponding to the *D* gene and the DH5α(λ_*c*I857_ΔD::KanR) lysogen produced a 950 bp band corresponding to the *kan* gene. We then evaluated the progeny production of both recombinant phages by transforming both lysogens with a pD_λ_ plasmid expressing a wild-type *D* gene regulated under a P_*tac*_ promoter and then inducing the prophages by changing the growth temperature condition from 32 to 42 °C for 15 min. We were not able to observe viral particles from the induction of the λΔD::KanR prophage, but normal production of viral particles was observed after λΔD prophage induction (Fig. 1c).

### Construction and evaluation of fusion proteins D_λ_-ZE_DIII_-6xHis, D_λ_-GFP-L-6xHis, D_λ_-GFP, D_λ_-GFP-L, and pD_λ_-TD-6xHis

To establish the phage display system, we fused the DNA fragments coding for the domain III of the Envelope protein E of ZIKV (ZE_DIII_; from residue S405 to D586) with the 3′-terminal of the *D*_*λ*_ gene, using a linker fragment coding for 18-amino acids between these two proteins (D_λ_-ZE_DIII_-L-6xHis) (Supplemental Fig. S1a). The pD_λ_-ZE_DIII_-L was used as a backbone for further constructions, changing either the linker (pD_λ_-ZE_DIII_−6xHis), or the nucleotides coding for the different cargo proteins (pD_λ_-GFP-L-6xHis, pD_λ_-ZE_DIII_, pD_λ_-GFP, and pD_λ_-TD-6xHis; Supplemental Fig. S1b). The D_λ_-TD-6xHis fusion was designed to display three different epitopes from ZE_DIII_ fused in tandem and separated by short linkers (Supplemental Fig. S1c).

In the subsequent experiments, we focused on the evaluation of the λ_evo_ platform displaying the D_λ_-ZE_DIII_−6xHis fusion protein, which was generated in a W3110 strain transformed with the corresponding plasmid. Trans production of the D_λ_-ZE_DIII_−6xHis protein was assessed by SDS-PAGE, in which a discrete band of 26.9 kDa was observed (Supplemental Fig. S3a). The fusion protein was verified by distinct immunodetection assays using an anti-6xHis antibody (Supplemental Fig. S3b), a commercial anti-ZE_DIII_ antibody, and anti-SI-D_λ_-ZE_DIII_−6xHis mouse serum (Supplemental Fig. S3c). However, the overproduction of D_λ_-ZE_DIII_−6xHis lead to the formation of inclusion bodies (Supplemental Fig. S3d); therefore, all evaluations were further performed under the basal protein production resulting from the transcriptional leakage of the P_*tac*_ promoter.

### Generation of phage λ_evo_ by directed evolution

**Fig. 2:**
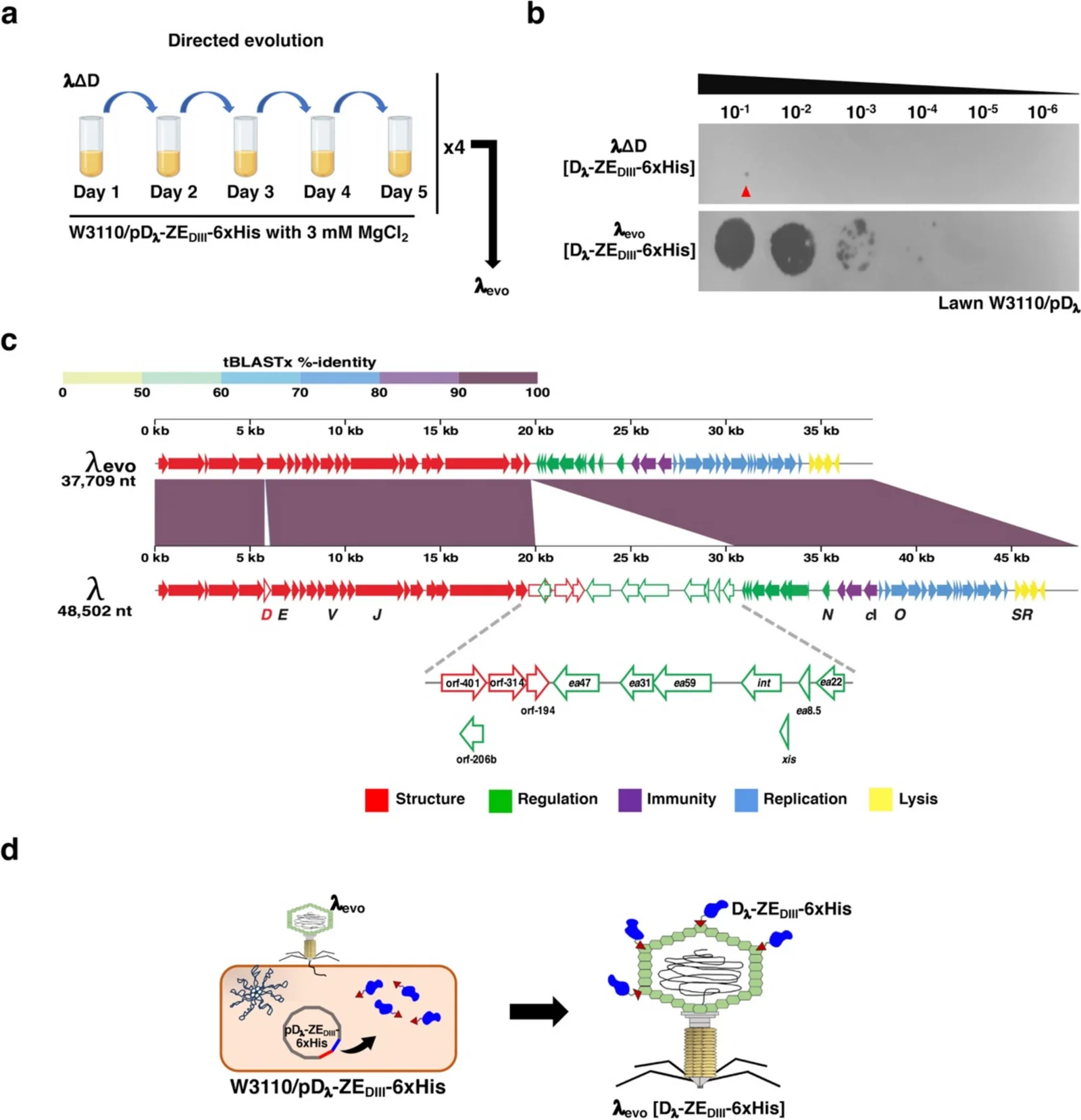
The phage display system in λ_evo_. **a** Design of the directed evolution assay to enhance decoration of phage λΔD with D_λ_-ZE_DIII_−6xHis. Phage λΔD was subjected to sequential infection-lysis daily rounds by adding fresh medium and bacterial cultures (see “Materials and methods” section). After four cycles of 5 days of directed evolution, the novel phage λ_evo_ was obtained. **b** Titration of λΔD and λ_evo_ complemented in trans with the fusion protein D_λ_-ZE_DIII_−6xHis. The viral titer of λΔD obtained before the directed evolution approach was ~ 1 × 10^3^ PFU/ml; the resulting phage λ_evo_ showed a 3000-fold increase in its viral titer. **c** Genome alignment of phages λ_wt_ and λ_evo_ using the ViPTree server (https://www.genome.jp/viptree/). Besides the deletion of *D* gene, the phage λ_evo_ harbored a deletion of the *b* region from nucleotides 19,903 to 30,549 (indicated by dotted lines). This region comprises about 22% of its genome and includes non-essential genes, such as *int* and *xis*. Clusters of genes are colored according to their functional class. **d** Schematic representation of the strategy used to obtain phage λ_evo_ decorated with the D_λ_-ZE_DIII_−6xHis fusion protein. The phage λ_evo_ infects a W3110 strain producing the fusion protein from plasmid pD_λ_-ZE_DIII_−6xHis, resulting in phage λ_evo_ virions displaying D_λ_-ZE_DIII_−6xHis on their capsids

The amount of phage λΔD obtained from a liquid lysate of W3110/pD_λ_-ZE_DIII_−6xHis was low (~ 3.3 × 10^3^ PFU/ml), according to the plaque assays (Fig. 2b and Supplemental Fig. S2). To enhance the phage titer, we undertook a directed in vitro evolution assay, carefully selecting phages that were adapted to low concentrations of Mg^2+^ (3 mM MgCl_2_), in the bacterial host producing the D_λ_-ZE_DIII_−6xHis protein. Divalent cations (e.g., Mg^2+^, Ca^2+^) stabilize virion structures possibly by neutralizing the negative charges of DNA; therefore, a reduced availability of such cations in liquid lysates contributed to maintain a selective pressure favoring phages mainly stabilized by the fusion protein (Fig. 2a). After 20 days (20 cycles of dilution), we observed an increase and stabilization of the phage titer at ~ 1 × 10^7^ PFU/ml (Fig. 2b). Only four viable phage lineages were generated from six independent biological replicates of directed evolution, and the one with the highest viral titer was selected and named λ_evo_.

We sequenced the λ_evo_ genome to assess whether this evolved phage accumulated genetic changes that could lead to an improved titer. The viral proteomic tree server (ViPTree v4.0) (Nishimura et al. 2017) was used to perform the comparative genomic analysis using the phage λ genome as a reference (Fig. 2c). The phage λ_evo_ harbors a 10,793 bp deletion, which represents approximately 22% of the λ genome. The deletion is in the λ *b* region, corresponding to positions 19,993 to 30,548 according to the reference genome reported in GenBank (accession number J02459), and disrupted the open reading frame 401 (orf-401) and removed six non-essential genes (including *int* and *xis*) (Fig. 2c). The display system subsequently used in this work was obtained from liquid lysates of phage λ_evo_ in a W3110 strain complemented with a D_λ_ derivative fusion protein (e.g., D_λ_-ZE_DIII_−6xHis). The pool of fusion protein generated by transcriptional leakage was sufficient to stabilize phage λ_evo_ [D_λ_-ZE_DIII_−6xHis] capsids (Fig. 2d).

### Optimization of phage display conditions

**Fig. 3:**
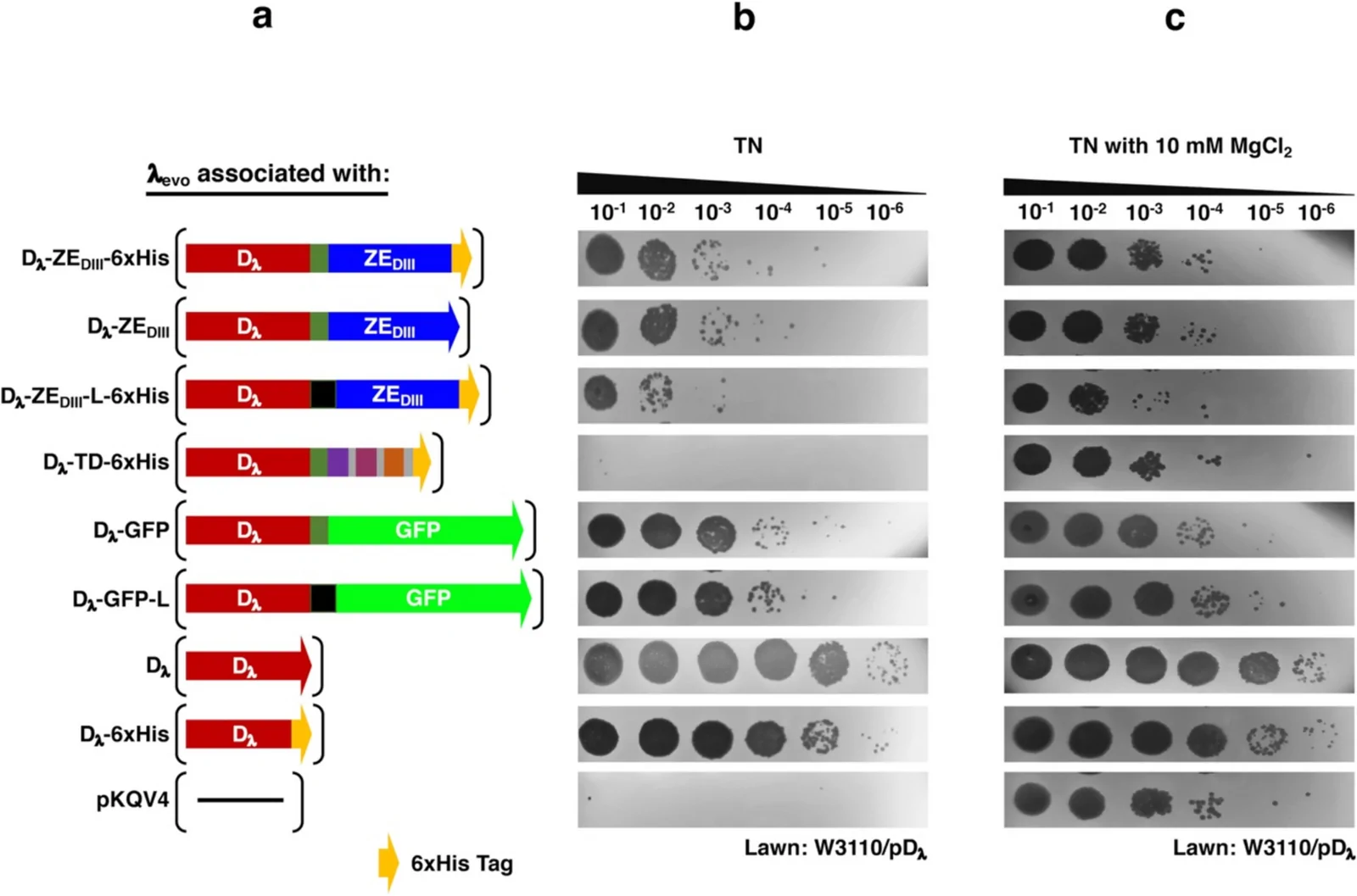
Production of λ_evo_ phages decorated in vivo with different fusion proteins. **a** Schematic representation of the plasmid constructs used for the expression of fusion proteins. All constructs contain the P_*tac*_ promoter. The coding sequence for D_λ_ and ZE_DIII_ are represented by a red and blue rectangle, respectively. The linkers between these two proteins are represented by a green square (short linker) or a black square (long linker), and the yellow arrowhead represents the 6xHis tag. For the tandem construct, the coding sequence from D_λ_ was fused with sequences corresponding to three different epitopes of the domain III of the E_ZIKV_ protein, which were adjacently positioned to each other. **b** Production of λ_evo_ phages stabilized with the different fusion proteins expressed from the plasmids described above. Serial dilutions were prepared with TN buffer and spotted onto a W3110/pD_λ_ lawn. The titration lanes correspond to the plasmid illustrations on the left panel. **c** Stabilization effect of Mg^2+^ in the total production of λ_evo_ phages stabilized with different fusion proteins. Serial dilutions of phages were prepared with TN buffer supplemented with 10 mM MgCl_2_. Serial dilutions were spotted onto a W3110/pD_λ_ bacterial lawn. All experiments were performed in triplicate

Using the λ_evo_ phage, the display of different fusion proteins was evaluated (Fig. 3a). Generally, when wild type λ phage (λ_wt_ or equivalent) displays D_λ_, it maintains a high viral titer, independent to the Mg^2+^ concentration (Fig. 5; Mikawa et al. 1996; Nicastro et al. 2013). Phages decorated with different fusion proteins were evaluated in the absence (TN buffer) and presence of Mg^2+^ (TN buffer with 10 mM MgCl_2_), to assess their stabilization (Fig. 3b and 3c). The titer of phages differed among all the evaluated peptides (Fig. 3b). The display of D_λ_-ZE_DIII_ fusions produced a low viral titer, ranging from 2 × 10^6^ to 6 × 10^7^ PFU/ml. Only tenfold of difference in titer was observed between the D_λ_-ZE_DIII_−6xHis short linker (GGSGALE) and the D_λ_-ZE_DIII_-L-6xHis long linker (GGSGALENLYFQSPPTPTS) fusion proteins; the presence or absence of the 6xHis tag did not produce any differences in the display.

Regarding the other fusion proteins, despite GFP is larger than ZE_DIII_, the D_λ_-GFP fusions yielded a higher phage titer (1.0 × 10^8^ to 1.7 × 10^8^ PFU/ml) with both linkers. Interestingly, the shortest fusion protein (D_λ_-TD-6xHis) failed in the display, as revealed by the lack of stable viral titer (Fig. 3b). The D_λ_ and D_λ_−6xHis display had a viral titer equivalent to that of λ_wt_ (2.7 × 10^9^ to 1.6 × 10^10^ PFU/ml), as expected.

**Fig. 4:**
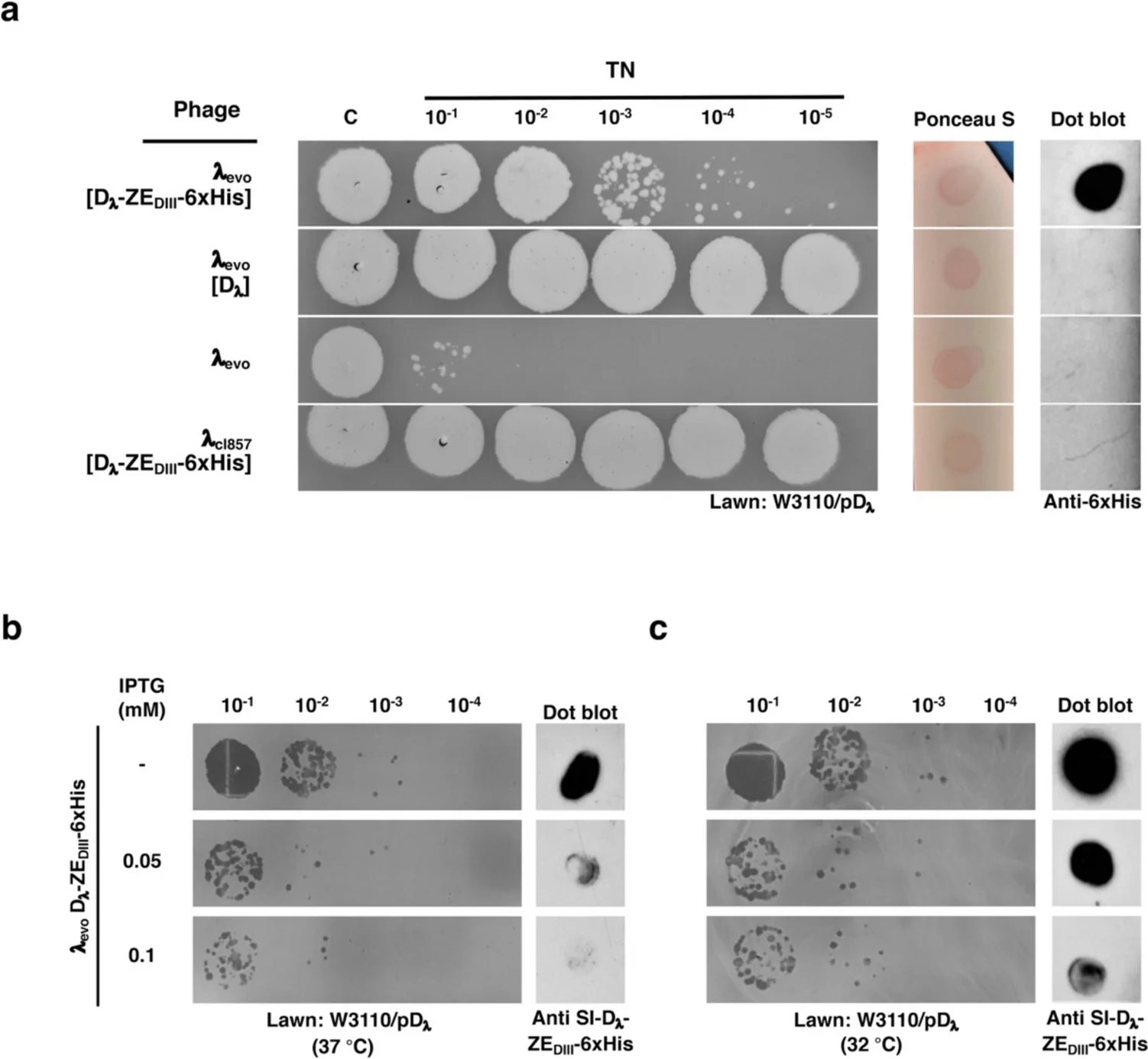
Evaluation of the D_λ_-ZE_DIII_−6xHis display in phage λ_evo_ by dot blot. **a** Plaque assay comparison of concentrated phage λ_evo_ lysates differentially decorated in vivo with D_λ_-ZE_DIII_−6xHis (λ_evo_ [D_λ_-ZE_DIII_−6xHis), D_λ_ (λ_evo_ [D_λ_]) or none. Serial dilutions were spotted onto W3110/pD_λ_ lawns, for each phage lysate as indicated at the left. The lysate from phage λ_wt_ in W3110/pD_λ_-ZE_DIII_−6xHis strain was used as a control. The right panels show the immunodetection of the 6xHis tag on lysate spots dropped on a nitrocellulose membrane, using an anti-6xHis antibody. The total protein amount on each spot was previously normalized, as shown by the Ponceau S staining. **b** Effect of IPTG in the production of phage λ_evo_ decorated with fusion proteins. Left panel: plaque assay comparison of phage lysates in which distinct concentrations of IPTG (as shown at the left) were used to induce the plasmid expression of the fusion protein at 37 °C. Right panel: dot blot immunodetection with anti-SI-D_λ_-ZE_DIII_−6xHis, using spots with the same amounts of phage particles, for each induction condition. **c** Effect of a lower temperature (32 °C) in the production of phage λ_evo_ decorated with the fusion protein, at distinct IPTG concentrations. Right panel: Dot blot immunodetection using anti-SI-D_λ_-ZE_DIII_−6xHis with similar amounts of phages in each spot. All serial dilutions in the assay were plated on W3110/pD_λ_ lawns

In order to evaluate the presence of D_λ_-ZE_DIII_−6xHis protein on λ_evo_ phage capsids, λ_evo_ phage lysates were firstly precipitated with 16% PEG-8000 and resuspended in TN buffer (10 mM Tris–HCl, 100 mM NaCl, pH = 7.8) with 10 mM MgCl_2_. The concentrated λ_evo_ decorated in vivo with the D_λ_-ZE_DIII_−6xHis fusion protein (λ_evo_ [D_λ_-ZE_DIII_−6xHis]), with wild-type D_λ_ (λ_evo_ [D_λ_]), and without fusion proteins, were used for infection assays. The stabilization of phage titer by decoration was assessed by serial dilutions in TN buffer (10 mM Tris–HCl, 100 mM NaCl, pH = 7.8), spotted on W3110/pD_λ_ lawns (Fig. 4a). The number of stable phages was observed by plaque infection assays; noteworthy, the λ_evo_ phages could be stabilized only with the Mg^2+^ traces from the resuspension buffer, generating 4 × 10^4^ PFU/ml.

To determine the presence of D_λ_-ZE_DIII_−6xHis on phage capsids, we initially immunodetected the 6xHis tag by dot blot, and the only signal observed corresponded to the λ_evo_ phages decorated with D_λ_-ZE_DIII_−6xHis (Fig. 4a). Notably, there was no signal in the λ_cI857_ sample obtained from a W3110/pD_λ_-ZE_DIII_−6xHis lysate. Dot blot samples were normalized by quantification of total protein by Bradford assays. The Ponceau-S stain confirmed similar amounts of protein between all samples (Fig. 4a). The amount of total viable phages stabilized with magnesium remained similar in λ_evo_ [D_λ_-ZE_DIII_−6xHis] and λ_evo_ samples (Supplemental Fig. S4a), and no viral production could be observed when these phages were titrated in a wild-type W3110 lawn with no production of the D_λ_ protein (Supplemental Fig. S4b).

We evaluated the effect of IPTG concentration and temperature on phage production in the D_λ_-ZE_DIII_−6xHis display platform. Contrary to our expectations, a lower viral titer was produced when the IPTG inducer was increased (Fig. 4b, c). We observed a decrease in phage production at 37 °C by 1 or 2 folds; however, incubation at 32 °C enhanced the amount of protein displayed in the capsid of λ_evo_, according to dot blot assays (Fig. 4b, c).

### λ_evo_in vitro decoration with D_λ_-ZE_DIII_-6xHis protein

**Fig. 5:**
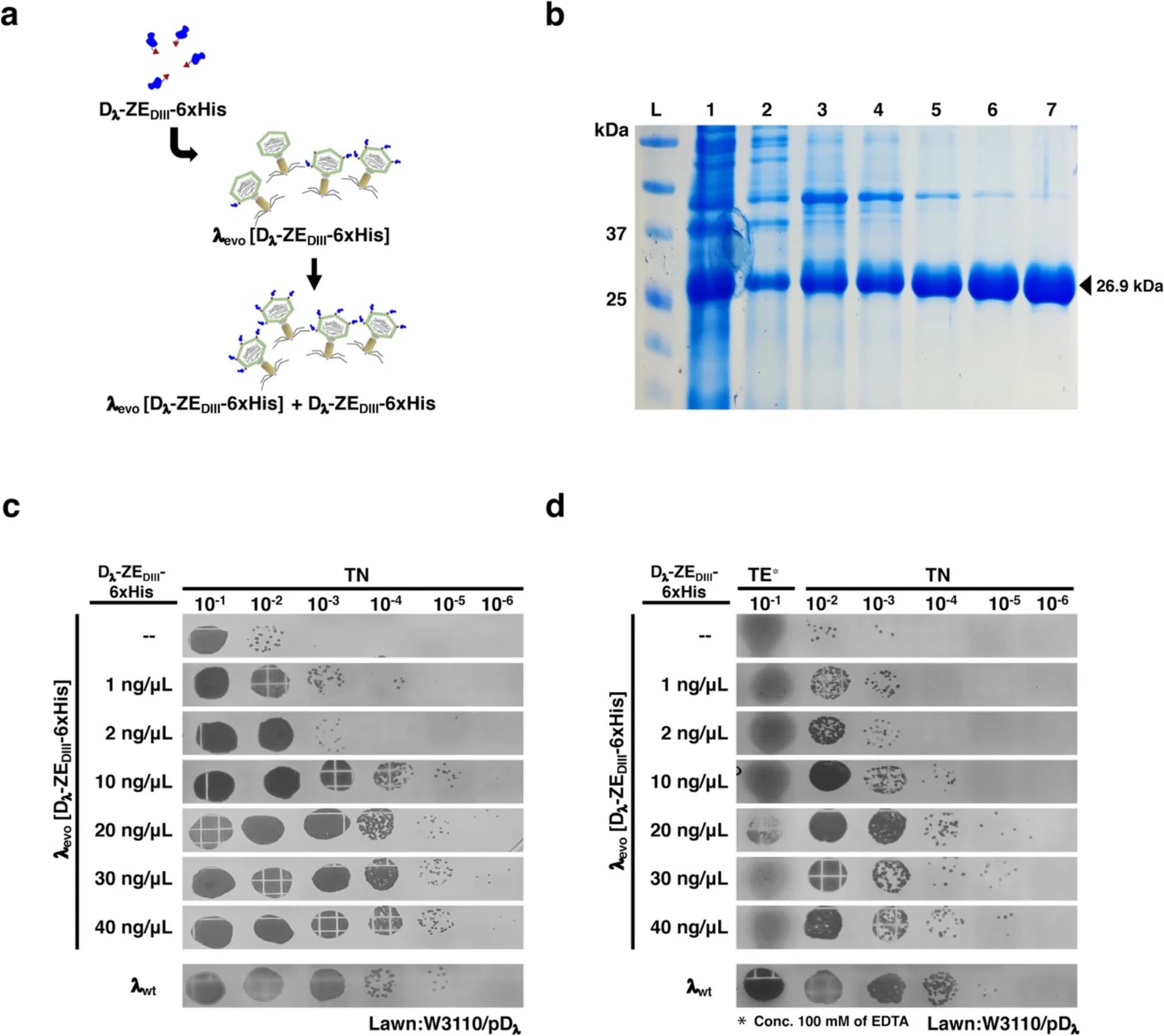
In vitro decoration of phage λ_evo_ [D_λ_-ZE_DIII_−6xHis]. **a** Cartoon of the in vitro decoration strategy. The purified protein D_λ_-ZE_DIII_−6xHis was added to phage λ_evo_ [D_λ_-ZE_DIII_−6xHis] to obtain the phage λ_evo_ [D_λ_-ZE_DIII_−6xHis] + D_λ_-ZE_DIII_−6xHis, which displayed a substantial increase in the number of protein fusion decorations. **b** 16% SDS-PAGE analysis of the purification process of the D_λ_-ZE_DIII_−6xHis protein. Gel lanes correspond to the molecular weight ladder (L), total protein extract (1), fractions of the successive washes with 8 M urea pH = 6.4 (2, 3, and 4), and fusion protein elution fractions with 8 M urea pH = 4.5 (5, 6, and 7). The fusion protein calculated size is ~ 26.9 kDa. **c** Plaque assay comparing in vitro decoration with different concentrations of purified D_λ_-ZE_DIII_−6xHis protein and its effect in phage stabilization. Serial dilutions of in vitro decorated λ_evo_ [D_λ_-ZE_DIII_−6xHis] phage preparations were spotted onto W3110/pD_λ_ lawns; the concentrations of the purified fusion protein used in each preparation are indicated at the left. Ten ng/μl of purified protein were sufficient to obtain a viral titer of λ_evo_ [D_λ_-ZE_DIII_−6xHis] + D_λ_-ZE_DIII_−6xHis similar to that of the λ_wt_ control (bottom). **d** Effect of 100 mM of initial EDTA in the efficiency of phage in vitro decoration. TE buffer was initially added to in vitro decorated phages using distinct amounts of purified proteins (as indicated at the left), and then, serial dilutions were prepared with TN and spotted onto W3110/pD_λ_ lawns. The maximum decoration obtained for the phage λ_evo_ [D_λ_-ZE_DIII_−6xHis] + D_λ_-ZE_DIII_−6xHis was observed with 20 ng/μl of purified fusion protein

To improve phage decoration display, in vitro addition of previously purified fusion proteins was tested (Fig. 5a and Supplemental Fig. S5a). We used both λ_evo_ and λ_evo_ [D_λ_-ZE_DIII_−6xHis] lysates and added D_λ_-ZE_DIII_−6xHis protein (Fig. 5b), which was purified under denaturing conditions and subsequently renatured. This approach increased the number of stabilized phages (Fig. 5c and Supplemental Fig. S5b), and the maximal threshold was observed when ~ 10 ng/ml of the recombinant protein were added to the lysates (λ_evo_ [D_λ_-ZE_DIII_−6xHis] + D_λ_-ZE_DIII_−6xHis; Fig. 5c), or ~ 20 ng/ml (λ_evo_ + D_λ_-ZE_DIII_−6xHis; Supplemental Fig. S5b); no increase in stabilized phages was further observed after adding more protein. To assess whether the presence of the D_λ_-ZE_DIII_−6xHis fusion protein stabilized the phage capsid, we tested the stability of λ_evo_ [D_λ_-ZE_DIII_−6xHis] + D_λ_-ZE_DIII_−6xHis and λ_evo_ + D_λ_-ZE_DIII_−6xHis in the presence of EDTA. The concentrated lysate was initially diluted 1:10 with TE buffer (100 mM EDTA), and subsequent dilutions without EDTA were prepared. We observed a slight decrease on the λ_evo_ [D_λ_-ZE_DIII_−6xHis] + D_λ_-ZE_DIII_−6xHis samples where the fusion protein was presented, and nearly to a tenfold reduction in the λ_evo_ + D_λ_-ZE_DIII_−6xHis phages. However, in the absence of purified protein, no viral particles could be observed for λ_evo_ after EDTA treatment (Supplemental Fig. S5c), thus indicating that decoration with the fusion protein stabilized the λ_evo_ phage capsids.

### Structural analysis of λ_evo_ [D_λ_-ZE_DIII_-6xHis] by transmission electron microscopy

**Fig. 6:**
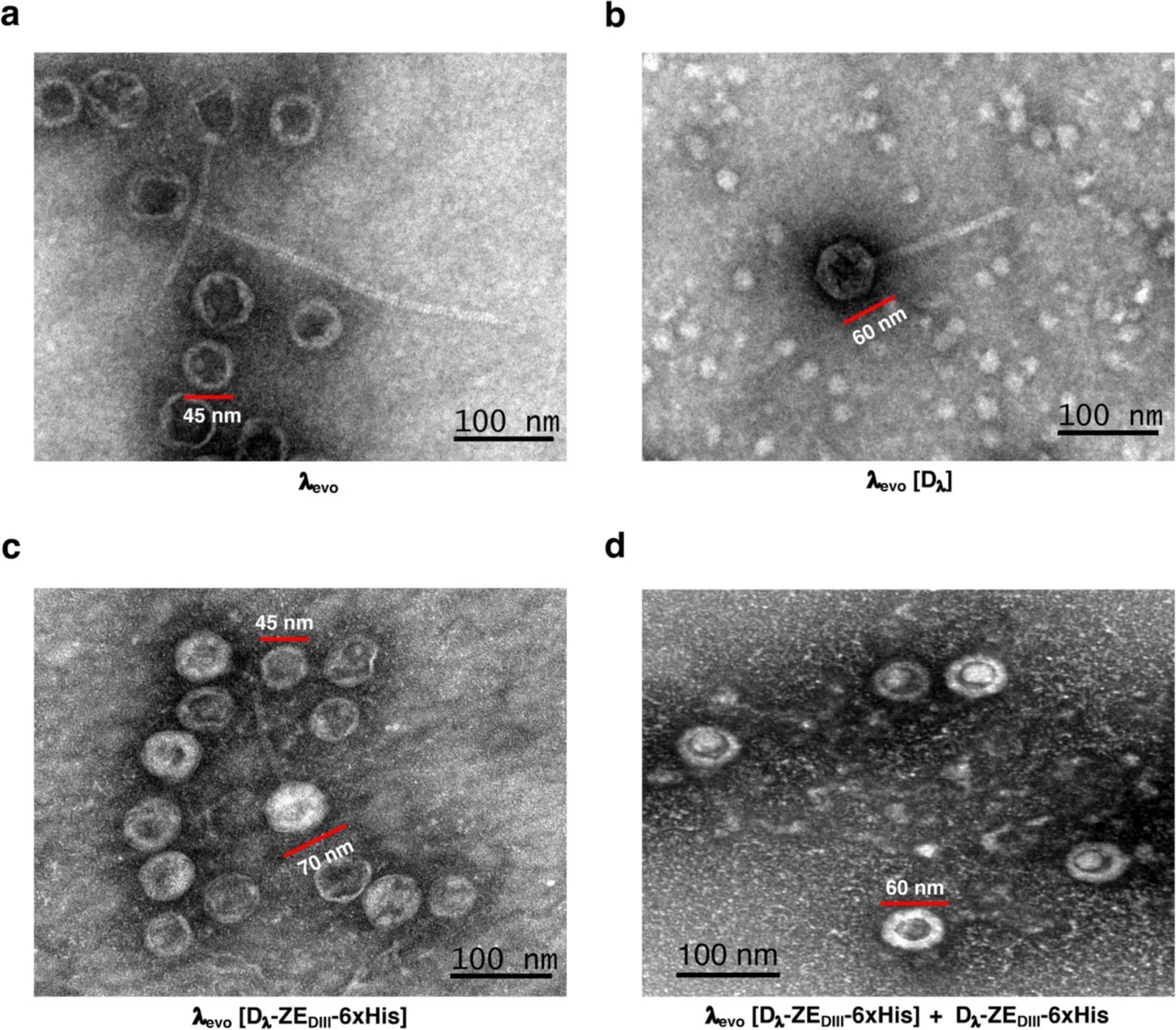
Transmission electron microscopy of λ_evo_ phages decorated through different strategies. **a** λ_evo_ without decoration showing empty pre-capsids measuring ~ 45 nm. **b** The λ_evo_ [D_λ_] morphology is similar to λ_wt_ phage, with a capsid size of approximately 60 nm. **c** In vivo decorated λ_evo_ [D_λ_-ZE_DIII_−6xHis] displaying a morphology of pre-capsids mixed with complete phages featuring larger capsids (~ 70 nm); protein aggregations are observed (white regions). **d** The in vivo/in vitro decorated λ_evo_ [D_λ_-ZE_DIII_−6xHis] + D_λ_-ZE_DIII_−6xHis phage is mainly found with a morphology consisting in highly decorated small capsids without tails; the light refraction generated by the viral envelope formed around the capsid is increased (thicker), compared to phages without the addition of protein

Bacteriophage lysates were concentrated with PEG-8000 and then subjected to a CsCl gradient; five bands of migrating phages (i.e., different densities) could be observed in the λ_evo_ [D_λ_-ZE_DIII_−6xHis] samples (Supplemental Fig. S6a). Bands number three and two were selected for the phages λ_evo_ [D_λ_] and λ_evo_ (Supplemental Fig. S6d and S6e), respectively; the phages corresponding to these different bands were analyzed by transmission electron microscopy (TEM) to visualize their morphologies. The B3 band of λ_evo_ [D_λ_-ZE_DIII_−6xHis] presented a population of phages with different capsid sizes, ranging from 45 to 70 nm in diameter (Fig. 6c). In the case of phage λ_evo_ [D_λ_-ZE_DIII_−6xHis] + D_λ_-ZE_DIII_−6xHis (in vitro decoration), bright halos were observed on the capsids, which were out of the ordinary, with a size of 45 nm (Fig. 6d); phage λ_evo_ had a high prevalence of pre-capsids with a diameter of 45 nm (Fig. 6b). As expected, phage λ_evo_ [D_λ_] presented a homogeneous morphology, like wild-type phage λ (Fig. 6a).

### Immune response against λ_evo_ [D_λ_-ZE_DIII_-6xHis]

**Fig. 7:**
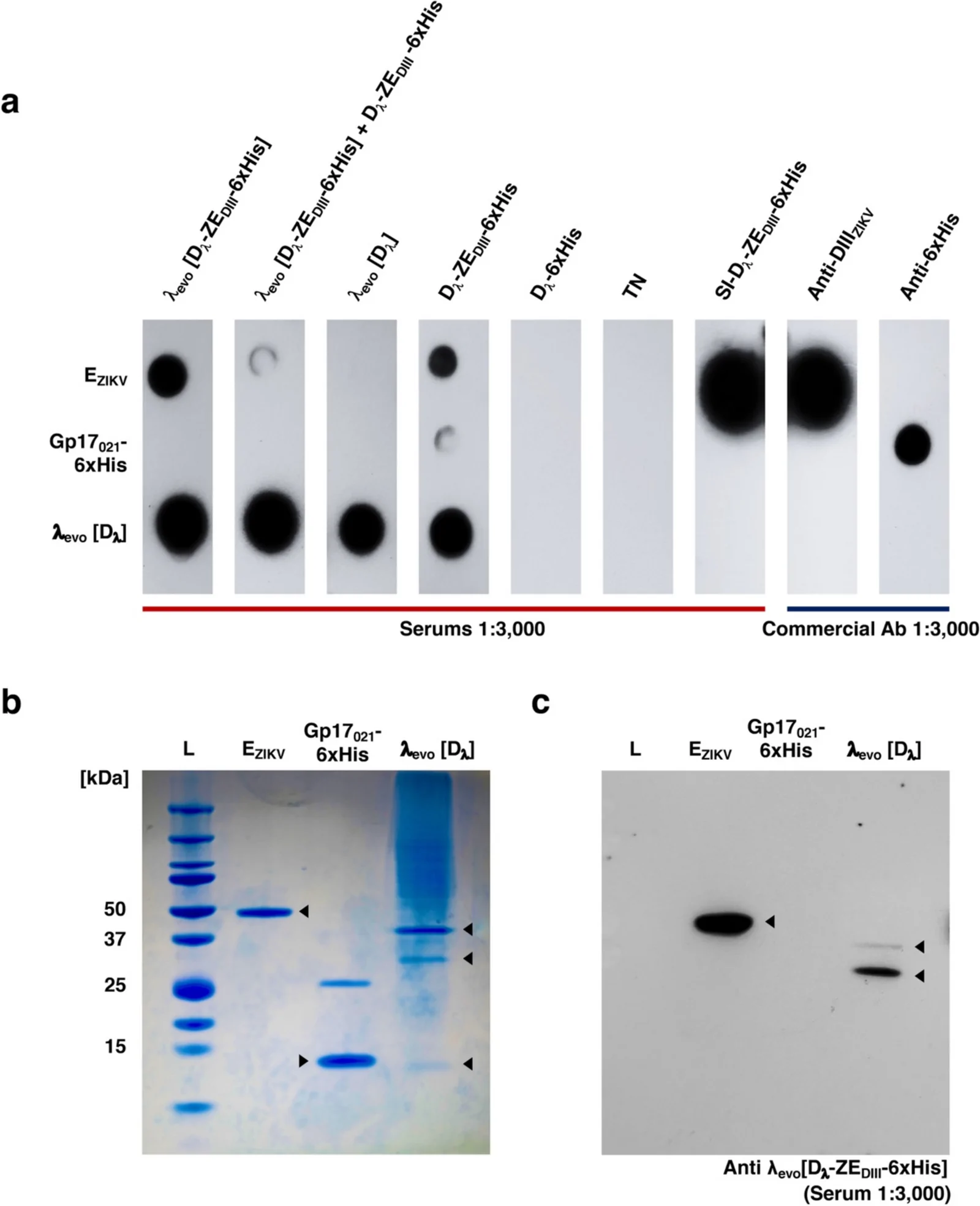
Immunodetection of E_ZIKV_ with immunized mice sera. **a** Dot blot immunodetection of purified proteins E_ZIKV_, Gp17-6xHis and liquid λ_evo_ [D_λ_] lysate samples (as indicated at the left of the figure), using the sera obtained from the immunization assays or commercial antibodies (as indicated by top labels). The E_ZIKV_ protein is strongly recognized by the SI-D_λ_-ZE_DIII_−6xHis serum and by the anti-ZE_DIII_ antibody. Lower signal is observed by using sera obtained from λ_evo_ [D_λ_-ZE_DIII_−6xHis], λ_evo_ [D_λ_-ZE_DIII_−6xHis] + D_λ_-ZE_DIII_−6xHis, and D_λ_-ZE_DIII_−6xHis immunizations. The histidine tag of the Gp17-6xHis protein was recognized by the D_λ_-ZE_DIII_−6xHis serum and the anti-6xHis antibody. Strong recognition of λ_evo_ [D_λ_] was observed with almost all the sera obtained from inoculation of differentially decorated λ_evo._ TN serum did not recognize any of the samples. All antibodies were used in 1:3,000 dilutions. **b** The integrity of all protein samples was evaluated by SDS-PAGE stained with Coomassie blue. E_ZIKV_ ectodomain (44.6 kDa) and Gp17-HisTag (11.4 kDa) were observed as discrete bands. λ_evo_ [D_λ_] proteins E_λ_ (38.2 kDa), V_λ_ (25.8 kDa) and D_λ_ (11.6 kDa) were also observed. **c** Immunodetection of the samples displayed in **b** using the λ_evo_ [D_λ_-ZE_DIII_−6xHis] serum shows recognition of E_ZIKV_, E_λ_, and V_λ_

Production of antibodies against the decorated phages was carried out by inoculating ~ 1 × 10^6^ PFU/ml of the different phages displaying D_λ_-ZE_DIII_ (λ_evo_ [D_λ_-ZE_DIII_−6xHis] and λ_evo_ [D_λ_-ZE_DIII_−6xHis] + D_λ_-ZE_DIII_−6xHis) and 1 × 10^6^ PFU/ml of λ_evo_ [D_λ_] into 14-day-old BALB/c mice. As control, 1 μg of purified D_λ_-ZE_DIII_−6xHis and D_λ_ proteins were respectively inoculated without adjuvant. The obtained sera were tested for immunodetection of E_ZIKV_, Gp17_021_−6xHis (Valencia-Toxqui et al 2023) and a liquid lysate sample of λ_evo_ [D_λ_] by dot blot (Fig. 7a). Recognition of E_ZIKV_ protein could be observed in a serum obtained from the inoculation of D_λ_-ZE_DIII_ (λ_evo_ [D_λ_-ZE_DIII_−6xHis] and λ_evo_ [D_λ_-ZE_DIII_−6xHis] + D_λ_-ZE_DIII_−6xHis), and in serum obtained from the inoculation of the fusion protein alone. From these, only the latter could detect the 6xHis tag of the Gp17_021_−6xHis protein, indicating a low recognition of the 6xHis portion of the fusion protein. We observed that almost all sera contained antibodies against λ_evo_ [D_λ_]. To ensure the integrity of all the protein samples used for dot blot and Western blot analysis, these were evaluated by SDS-PAGE and observed by Coomassie staining (Fig. 7b). The serum induced with λ_evo_ [D_λ_-ZE_DIII_−6xHis] was also evaluated by Western blot (Fig. 7c); this serum was able to detect the recombinant E_ZIKV_ protein (44.6 kDa) (Fig. 7c) and the ZIKV lysate (55 kDa) (Supplemental Fig. S7a) and can also recognize the E_λ_ (38.2 kDa) and V_λ_ (25.8 kDa) proteins in a λ_evo_ [D_λ_] sample. We therefore show that the phage display system generated in this work can induce the production of specific antibodies against ZIKV.

## Discussion

In recent years, studies of phages and their molecular components have taken great importance in technological and biomedical applications, as well as in basic research. Bacterio-viruses as bacterial pathogens have great potential in the fight against “super-bugs” (Pires et al. 2020; Pirnay et al. 2021). Among these applications, phage display was conceived to generate peptide libraries to test their affinity to different ligands, allowing easy selection and amplification of the peptides. However, due to their nature as peptide carriers, phage display can induce an immune response when used as a vaccine (Hess and Jewell 2020). A display phage was expected to serve as a natural immune-stimulant, being innocuous, highly stable and versatile, leading to cost-effective preparations (Cano et al. 2017; Palma 2023). In this study, we aimed to generate an efficient phage display platform that can eventually be used as an immunogen carrier. We selected phage λ due to its flexibility to display peptides of different sizes (Zucconi et al. 2001) and the high display density of large peptides, compared to filamentous phage platforms such as M13 (Gupta et al. 2003; Vaccaro et al. 2006).

To develop our system, we carefully selected the D_λ_ protein to generate different fusion proteins for display (Supplemental Fig. S1). This protein is known for its ability to improve the solubility of recombinant proteins in *E. coli*, and it was chosen due to its potential to generate a high-density display (up to ~ 370 fusion proteins/virion) (Forrer and Jaussi 1998; Zucconi et al. 2001). Furthermore, C-terminal end fusions allow the formation of disulfide bonds that are crucial for the correct folding and function of several proteins (Gupta et al. 2003; Hayes et al. 2010). The use of this type of fusion has also shown a higher number of proteins displayed on the phage capsid than N-terminal fusions (Vaccaro et al. 2006; Pavoni et al. 2013). However, we were aware that phage λ tends to accumulate mutations when the desired protein is engineered in cis, which often results in the abolition of the production of the fusion protein (Vaccaro et al. 2006). This led us to a design in which the fusion proteins were expressed in trans, in order to ensure the stability and functionality of our platform.

We have previously evaluated display by using a λ_cI857_ platform without modifications in the *D*_λ_ gene. However, this approach yielded no display of fusion protein when D_λ_-ZE_DIII_-L-6xHis was supplemented (Supplemental Fig. S8), due to higher affinity of D_λ_ for λ capsids, outcompeting the fusion protein (Nicastro et al. 2013). This encouraged us to evaluate the efficiency of the display using a λΔD platform. As expected, phage λΔD generated poor quantities of stable phages when complemented with fusion proteins (Supplemental Fig. S2), because the wild-type D_λ_ is essential to stabilize the genome contained inside the phage λ capsids (Sternberg and Weisberg 1977). Previous reports indicated that the display of recalcitrant proteins requires a two-gene strategy, one coding the fusion protein for display and another the wild-type D_λ_ protein for capsid stabilization, thus generating a divalent display (Mikawa et al. 1996; Santini et al. 1998; Mattiacio et al. 2011; Nicastro et al. 2013). Despite this, our main objective was to find the most efficient display in the absence of D_λ_ in phage λ. Previous reports have shown that in the absence of D_λ_, phage λ virions can be stabilized by divalent cations (e.g., Mg^2+^, Ca^2+^); however, this can only occur in genomes with less than 80% of the parental decoration (Sternberg and Weisberg 1977) (Fig. 3c and Supplemental Fig. S4a). Therefore, a directed evolution approach was applied to the λΔD phage, in order to obtain a mutant with improved decoration. This was carried out by sequential cycles of phage infection and lysis under D_λ_-ZE_DIII_−6xHis expression and low concentrations of MgCl_2_, which were imposed as selective pressures. Due to its close interaction with D_λ_, we initially expected some specific mutations in the E_λ_ gene. However, we observed a deletion encompassing ~ 22% of the λΔD genome instead (Fig. 2c), which accounted for an increased capacity to produce progeny decorated with D_λ_-ZE_DIII_−6xHis; this phage was named λ_evo_. The deleted region is involved in activating lysogenic/lysis pathways, and among other genes, *int* and *xis* were deleted, which is why λ_evo_ presents a clear plaque phenotype. Several large genome deletions have been described in the literature in phages lacking D_λ_ (Sternberg and Weisberg 1977), and several mutant phages were subsequently used in phage display (Eguchi et al. 2001; Zanghi et al. 2005). The phage λD1180 has been also used, and a deletion in the *nin* region and a shorter deletion in the *b* region were observed. The deletion observed in phage λ_evo_ improved the viral titer displaying different fusion proteins. These phages are stable under Mg^2+^-free conditions (Fig. 3b) and are even resistant to a challenge against EDTA (100 mM) (Fig. 5d). The resistance of phage λ to EDTA depends on the amount of fusion-protein occupying the D_λ_ sites on the phage capsid (Sternberg and Hoess 1995; Mikawa et al. 1996). Although λ_evo_ [D_λ_-ZE_DIII_−6xHis] showed a relatively low titer, it was stable, indicating that the display had a high efficiency.

Previously, Gupta et al. (2003) observed that display efficiency depends on the size of the fusion protein. We observed that the differences in stable viral titers obtained with different proteins (Fig. 3b) depend on the solubility of the fusion protein. For instance, the amount of phage obtained with the GFP protein was two log-units higher than that obtained with ZE_DIII_−6xHis, even though GFP is larger than ZE_DIII_ (Fig. 3b). The GFP protein has been reported to be soluble in *E. coli* (Waldo et al. 1999; Cha et al. 2000), while the ZE_DIII_ protein was highly insoluble, leading to the formation of inclusion bodies; we hypothesized that this could be due to the inefficient formation of the disulfide bonds in the *E. coli* background (e.g., between Cys308 and Cys339 of ZE_DIII_), which has been documented (Saejung et al. 2006; Yang et al. 2017; Cibulski et al. 2021). This insolubility was also observed in the D_λ_-ZE_DIII_−6xHis protein (Supplemental Fig. S3d). We therefore attempted to improve the yield of D_λ_-ZE_DIII_−6xHis by using *E. coli* strains with reduced inclusion body formation (e.g., the Origami strain), which have been shown to improve the solubility of EDIII_DENV_ (Saejung et al. 2006; Fahimi et al. 2014). However, the presence of prophage DE3 in such *E. coli* strains complemented the D_λ_ protein, making the strain incompatible to use with the λ_evo_ system (data not shown).

Interestingly, the shortest fusion protein (D_λ_-TD-6xHis) abolished the production of phages (Fig. 3d), which could not be restored even with directed evolution using this protein (data not shown). This suggests that the insolubility of this protein was the highest among all the fusion proteins we tested. Tandem epitopes tend to be highly insoluble when produced in *E. coli* (Colant et al. 2021; Jiang et al. 2021).

The production level of the fusion protein was determinant to achieve an adequate display. The amount of phage λ_evo_ [D_λ_-ZE_DIII_−6xHis] produced was inversely related on the inducer concentration (Fig. 4b, c). This was also related to the insolubility of D_λ_-ZE_DIII_−6xHis, as it was found in inclusion bodies when the amount of inducer was increased (Singh et al. 2015), thus decreasing the pool of soluble fusion protein available to stabilize λ_evo_ capsids. Similarly, cell growth temperature also affected protein solubility. Production of insoluble proteins at lower temperatures has been shown to increase their solubility (Saejung et al. 2006; Vera et al. 2007). Our results were consistent with this notion, where λ_evo_ [D_λ_-ZE_DIII_−6xHis] displayed higher decoration level at 32 °C than at 37 °C, and with different amount of the inducer (Fig. 4b, c). However, the viral titer did not change, indicating that these effects were not the result of changes in virion production.

Several groups have reported in vitro decoration approaches to display proteins on D_λ_-free phages by adding purified fusion proteins (Sternberg and Hoess 1995; Mattiacio et al. 2011; Chang et al. 2014). In vitro decoration allows the use of proteins produced in other systems with a more stable and accurate native conformation than the one obtained in *E. coli* systems (Mattiacio et al. 2011). Our in vitro decoration approach using purified [D_λ_-ZE_DIII_−6xHis] protein generated stable phages without MgCl_2_, improving viral titer in both λ_evo_ (without decoration) and in λ_evo_ decorated in vivo. Furthermore, these phages overcame an EDTA challenge with minimal losses of λ_evo_ [D_λ_-ZE_DIII_−6xHis] + D_λ_-ZE_DIII_−6xHis titer, and 1 log-unit loss of λ_evo_ + D_λ_-ZE_DIII_−6xHis protein, indicating that previous in vivo decoration enhances the uptake of soluble fusion protein (Fig. 5c, d and Supplemental Fig. S6c). However, the process for phage purification (e.g., Triton X-114 treatment or CsCl purification) washed away the fusion protein incorporated in vitro (Supplemental Fig. S9). This wash-out of the D_λ_-ZE_DIII_−6xHis fusion proteins does not occur upon in vivo decoration, suggesting that in vitro decoration may not be as efficient at fixing these proteins on the phage capsids.

One of the main challenges with the λ_evo_ display platform is the heterogeneity of phage populations displaying D_λ_-ZE_DIII_−6xHis: variable phenotypes were observed in the plaque assays (Figs. 2b and 3b and Supplemental Fig. S6b), the number of bands observed in the CsCl gradient differed to those of λ_evo_ [D_λ_] (Supplemental Fig. S6a and S6d), and the morphology observed by TEM was diverse (Fig. 6c and Supplemental Fig. S6c). This heterogeneity led to a reduction in the viral titer when phages were concentrated with PEG (data not shown), and to a decrease in the displayed protein after CsCl purification (Supplemental Fig. S6b). These concentration and purification processes are based on well-established protocols oriented to obtain highly purified phages. However, these techniques are often very aggressive towards phage particles, and this problem is aggravated in heterogeneous phage samples or phages with a low titer (Belleghem et al. 2017; Carroll-Portillo et al. 2021).

Finally, we evaluated the humoral response of BALB/c mice inoculated with different decorated phages and soluble proteins. We evaluated the sera of all the mice groups, and the most vigorous response was obtained against the D_λ_-ZE_DIII_−6xHis fusion protein when 40 μg of this purified protein was inoculated in a single dose with the adjuvant. However, antibodies generated with three doses of decorated phages λ_evo_ D_λ_-ZE_DIII_−6xHis and λ_evo_ [D_λ_-ZE_DIII_−6xHis] + D_λ_-ZE_DIII_−6xHis, were respectively observed, with a yield comparable to that obtained when a low concentration of D_λ_-ZE_DIII_−6xHis protein (1 μg) without adjuvant was used for immunization (Fig. 7a). These antibodies could recognize E_ZIKV_ in dot blot and Western blot assays (Fig. 7a, b). The low titer of λ_evo_ could explain the relatively low concentration of [D_λ_-ZE_DIII_−6xHis] antibodies produced, since it was not sufficient to induce an antibody yield against E_ZIKV_ similar to the one obtained with purified proteins inoculated with an adjuvant. Other works with lambda display platforms reported phage inoculations 10 to 100-fold higher (Gamage et al. 2009; Hayes et al. 2010; Mattiacio et al. 2011; Thomas et al. 2012; Arab et al. 2018; Barati et al. 2018; Razazan et al. 2019). However, our estimates of D_λ_-ZE_DIII_−6xHis protein in the CsCl-purified decorated phage λ_evo_ [D_λ_-ZE_DIII_−6xHis] (150 ng/dose) (Supplemental Fig. S10) are similar to those observed in other works at a 100 ng/dose (Mattiacio et al. 2011). This underscores the challenge of generating a strong antibody response, which we are determined to overcome.

Other factors accounting for the low antibody yields include the carrier-induced epitope suppression (CIES) event, in which a strong humoral response against the carrier could interfere with the reaction against the desired peptide (Jegerlehner et al. 2010). We suggest that CIES probably occurred in our assays, according to the high concentration of antibodies against the carrier phage that were observed in almost all preparations that could recognize E_λ_ and V_λ_ proteins (Fig. 7). The production of a high antibody titer against the main capsid proteins of inoculated phages has been observed in several works (Dąbrowska et al. 2014; Kaźmierczak et al. 2021). CIES has been previously observed in different phage carriers, but it can be avoided by using more significant doses of the immunogen. However, when CIES could not be avoided, phage display immunogens could be used as a booster of a protein priming strategy to enable a better humoral response (Jegerlehner et al. 2010; Mattiacio et al. 2011). CIES is an important event to consider because natural antibodies against phages have been previously reported in different systems, and these could interfere with the presented carriers even in phage therapy applications (Kaur et al. 2012; Dąbrowska et al. 2014; Jariah and Hakim 2019; Kaźmierczak et al. 2021; Dan et al. 2022). Comprehensive studies of the humoral response against this λ_evo_ display platform are warranted.

With this work, we demonstrate the possibility of generating a monovalent display platform in phage λ that decorates different sizes of proteins on its capsid. This platform achieved a high-density display on the phage capsids, which was suboptimal for a solid immunogenic induction. However, we tested proteins that are difficult to obtain in a soluble form in *E. coli* and therefore generated a low titer of phages; nevertheless, these could induce specific antibodies against ZIKV. The solubility of a protein is crucial for its successful display on phage, as soluble proteins are more likely to maintain their structural integrity and functional conformation. Consistently, the display of soluble protein GFP resulted in significant difference in viral titer performance compared with the display of insoluble protein ZE_DIII_−6xHis. We suggest that proteins with higher solubility might be more suitable candidates to be displayed on this platform, in order to generate a better immune response. The use of phage display is a powerful tool with potential use as a vaccine system, and finding the right conditions to optimize viral titers, fusion protein production and to improve immunogenicity is not a one-time task, but a constant challenge that must be refined for each of the antigens to be tested, underscoring the urgency and importance of our work.

## Supplementary Information

Below is the link to the electronic supplementary material.

Supplementary file1 (PDF 1217 KB)

## Data Availability

The datasets generated and analyzed during the current study are available from the corresponding author upon reasonable request.
