# Supplementary material for: A Lambda-evo (λevo) phage platform for Zika virus EDIII protein display
Source: Appl Microbiol Biotechnol. 2025 Jan 16;109(1):8. doi: 10.1007/s00253-024-13380-3 (PMC11739229; doi:10.1007/s00253-024-13380-3)
Supplement: Supplementary file 1 — Supplementary file1 (PDF 1217 KB) [file 253_2024_13380_MOESM1_ESM.pdf]

A Lambda-evo (λ_{evo}) phage platform for Zika virus E_{DIII} protein display

Honorio Negrete-Méndez[1], Guadalupe Valencia-Toxqui[2], Eva Martínez-Peñafiel[1], Oscar Medina-Contreras[3], Fernando Fernández-Ramírez[4], Edgar Morales-Ríos[5], Luis Janiel Navarro-González[5], Jesús M. Torres-Flores[6], and Luis Kameyama[1]*

[1.] Departamento de Genética y Biología Molecular, Centro de Investigación y de Estudios Avanzados del IPN, Av. Instituto Politécnico Nacional No. 2508, C.P. 07360, México City, México.
[2.] Department of Biology, Center for Phage Technology, Texas A&M University, College Station, TX, USA
[3.] Unidad de Investigación Epidemiológica en Endocrinología y Nutrición, Hospital Infantil de México Federico Gómez, Dr. Márquez No. 162, México City, México
[4.] Unidad de Genética, Hospital General de México. Dr. Balmis 148, C.P. 06726, México City, México.
[5.] Departamento de Bioquímica, Centro de Investigación y de Estudios Avanzados del IPN, Av. Instituto Politécnico Nacional No. 2508, C.P. 07360, México City, México.
[6] Laboratorio Nacional de Vacunología y Virus Tropicales, Escuela Nacional de Ciencias Biológicas del IPN, México City, México.

*Corresponding Author. Tel.: +52 555 747-3800 Ext.: 3340

E-mail: luisk@cinvestav.mx (Luis Kameyama).

Supplemental Table S1. Strains, bacteriophages, virus and plasmid constructions.
Supplemental Table S2. Oligonucleotides used in this study.
Supplemental Fig. S1. Schematic representation of plasmid constructs for fusion proteins expression
Supplemental Fig. S2. Overexpression of D_{λ}-ZE_{DIII}-6xHis in *E. coli*.
Supplemental Fig. S3. Evaluation of the λ_{cI857} display platform.
Supplemental Fig. S4. Expression of the wild-type D_{λ} protein stabilizes the distinct λΔD decorated phages.
Supplemental Fig. S5. Evaluation of phage viability using $MgCl_2$.
Supplemental Fig. S6. In vitro decoration of λ_{evo} phage.
Supplemental Fig. S7. Phage purification by CsCl gradient.
Supplemental Fig. S8. Resistance of λ_{evo} [D_{λ}-ZE_{DIII}-6xHis] and λ_{evo} [D_{λ}-ZE_{DIII}-6xHis] + D_{λ}-ZE_{DIII}-6xHis to Triton X-114 treatment.
Supplemental Fig. S9. Immunodetection of E_{ZIKV} in ZIKV lysates.
Supplemental Fig. S10. Estimation of the amount of D_{λ}-ZE_{DIII}-6xHis protein in different samples of λ_{evo} [D_{λ}-ZE_{DIII}-6xHis] phage.

Supplemental Table S1. Strains, bacteriophages and plasmid constructions.

***Escherichia coli* K-12 derivative strains**	**Genotype**	**Reference**
DH5α	*sup*E44 D(*lac*IZYA-*arg*F)U169 *deo*R (*Φ*80l*ac*ZDM15) *hsd*R17 *rec*A1 *end*A1 *gyr*A96 *thi*-1 *rel*A1	Hanahan 1983
DH5α(λ)	λ prophage	This work
DH5α(λΔD::Kan^R)	λΔD::Kan^R prophage	This work
DH5α(λΔD)	λΔD prophage	This work
W3110	F^- $λ^-$ *rph*$^-$	Bachmann 1972
JW2787	F- *Δ(araD-araB)567 ΔlacZ4787*(::*rrn*B-3) *λ ΔrecD744::kan rph-1 Δ(rhaD-rhaB)568 hsdR514*	Baba et al. 2006
SoluBL21†	F- *omp*T *hsd*SB (rB- mB-) *gal dcm* (DE3)	Deatherage et al. 2012

**Bacteriophages/viral strains**	**Genotype**	**Reference**
$λ_{wt}$	Lambda wild type	CSH Collection
$λ_{cI857}$	Lambda ts_{cI857}	CSH Collection
λΔD::Kan^R	$λ_{cI857}$ with a Kan^R cassette inserted in D site	This work
λΔD	$λ_{cI857}$ ΔD	This work
$λ_{evo}$	λΔD with a 10,793 bp deletion in b region	This work
ZIKV	Zika virus MEX_ENCB165P4	This work

**Plasmid**	**Relevant markers**	**Reference**
pJET1.2/blunt	*Eco471R* (Amp^R)	Thermo Fisher Scientific 2024
pJET-ZE_{DIII}-6xHis	pJET1.2/blunt with ZE_{DIII} coding sequence and 6xHis Tag (Amp^R)	This work
pJET-$D_λ$-6xHis	pJET1.2/blunt with $D_λ$ (Amp^R)	This work
pJET-$D_λ$	pJET1.2/blunt with $D_λ$ and 6xHis Tag (Amp^R)	This work
pJET-$D_λ$-ZE_{DIII}-L-6xHis	pJET1.2/blunt with $D_λ$-ZE_{DIII}-L fusion and 6xHis Tag	This work
pKQV4	P_{tac} promoter (Amp^R)	Strauch et al. 1989
p$D_λ$-ZE_{DIII}-6xHis	pKQV4 with $D_λ$-ZE_{DIII} fusion and 6xHis Tag (Amp^R)	This work
p$D_λ$-ZE_{DIII}-L-6xHis	p$D_λ$-ZE_{DIII}-6xHis with long linker (Amp^R)	This work
p$D_λ$-ZE_{DIII}	pKQV4 with $D_λ$-ZE_{DIII} (Amp^R)	This work
p$D_λ$-TD-6xHis	pKQV4 with $D_λ$ fused to 3 different epitopes from E_{ZIKV}, separated by short linkers and 6xHis Tag (Amp^R)	This work
p$D_λ$-GFP	pKQV4 with $D_λ$-GFP (Amp^R)	This work
p$D_λ$-GFP-L	p$D_λ$-GFP with long linker (Amp^R)	This work
p$D_λ$	pKQV4 with $D_λ$ (Amp^R)	This work
p$D_λ$-6xHis	pKQV4 with $D_λ$ and 6xHis Tag (Amp^R)	This work
pKD46	pBAD promoter *exo beta gam* genes (Cm^R)	Datsenko and Wanner 2000
pE-FLP	pBAD promoter *flp* gene (Cm^R)	Datsenko and Wanner 2000
pRSET-ZIKV/E_S	pRSET-A with E_{ZIKV} coding sequence corresponding to residues 1 to 409 (Amp^R)	This work

† The SoluBL21 strain contains uncharacterized mutations which were obtained through special selection criteria.

Supplemental Table S2. Oligonucleotides used in this study.

Primers	Sequence 5´ 3´	Use in this study
D_Recomb-Fw	cgttaacgatttgctgaacacaccagtgtaagggatgttttgtgtaggct ggagctgcttcg	λΔD construction by recombineering
D_Recomb-Rv	aaaaagccgcacaggcggcctttagtgatgaagggtaaagaattcc ggggatccgtcgacc	λΔD construction by recombineering
D_STOP-SD-Fw	**GAATTC**tgaggagaaaaaatgacgagcaaagaaac	Construction of pD_{λ}
D_STOP-Rv	**AAGCTT**ttaaacgatgctgattg	Construction of pD_{λ}
D-Fw	g**GAATTC**atgacgagcaaagaaac	Construction of pD_{λ}-6xHis and amplification of D_{λ}-ZE_{DIII}-6xHis
D_HisTag-Rv	c**AAGCTT**agtgatggtgatggtgatgaccgctgccaacgatgctg attgccgttc	Construction of pD_{λ}-6xHis
E_{ZIKV}(DIII)-Fw	**ACTAGT**gataaacttagattgaagggcgtgtcatactccttgtgta ccgcag	Construction of pJET-ZE_{DIII}
E_{ZIKV}-Rv	gagctc**AAGCTT**agtgatggtgatggtgatgtkattttccaatggtg ctg	Construction of pJET-ZE_{DIII} and amplification of F2
D_Link-Fw	ggaattcatgacgagcaaagaaacctttacccattaccagccgcag	Amplification of F1
Link(*Xho*I)-Rv	gctctgaaaatacaggttctcgagcgcaccgctaccgccctgcagta aaacgatgctg	Amplification of F1
Link(*Xho*I)-Fw	gagaacctgtattttcagagcccacccacgccgacctcgtctagaga taaacttagattgaag	Amplification of F2
2Step-Rv	gagttc**AAGCTT**agtgatggtg	Amplification of D_{λ}-ZE_{DIII}-6xHis
Mod2Fw	gagaagaagatcacccaccactggcacaggag	Site-directed mutagenesis to correct nonsynonymous mutations
Mod2Rv	ctcctgtgccagtggtgggtgatcttcttctc	Site-directed mutagenesis to correct nonsynonymous mutations
Mod3Fw	ctgaaagcactgagaactctaagatgatgctggaac	Site-directed mutagenesis to correct nonsynonymous mutations
Mod3Rv	gttccagcatcatcttagagttctcagtgctttcag	Site-directed mutagenesis to correct nonsynonymous mutations
LinkFlex-1	gggcggtagcggcgcgctggaat	Construction of pD_{λ}-ZE_{DIII}-6xHis
LinkFlex-2	ctagattccagcgcgccgctaccgccctgca	Construction of pD_{λ}-EZ_{DIII}-6xHis
Xtag-Fw	tggaaaatcataagcttctgttttggcggatgagagaagattttcag	Site-directed mutagenesis to remove 6xHisTag from D_{λ}-ZE_{DIII}- 6xHis
Xtag-Rv	cagaagcttatgattttccaatggcgctgccactcctgtgccagtggt	Site-directed mutagenesis to remove 6xHisTag from D_{λ}-ZE_{DIII}- 6xHis
GFP-Fw	**TCTAGA**atgagtaaaggagaagaact	Construction of pD_{λ}-GFP-L and pD_{λ}-GFP
GFP-Rv	**AAGCTT**ctatttgtatagttcatcca	Construction of pD_{λ}-GFP-L and pD_{λ}-GFP

Sequences in bold capital letters represent the restriction enzyme sites.

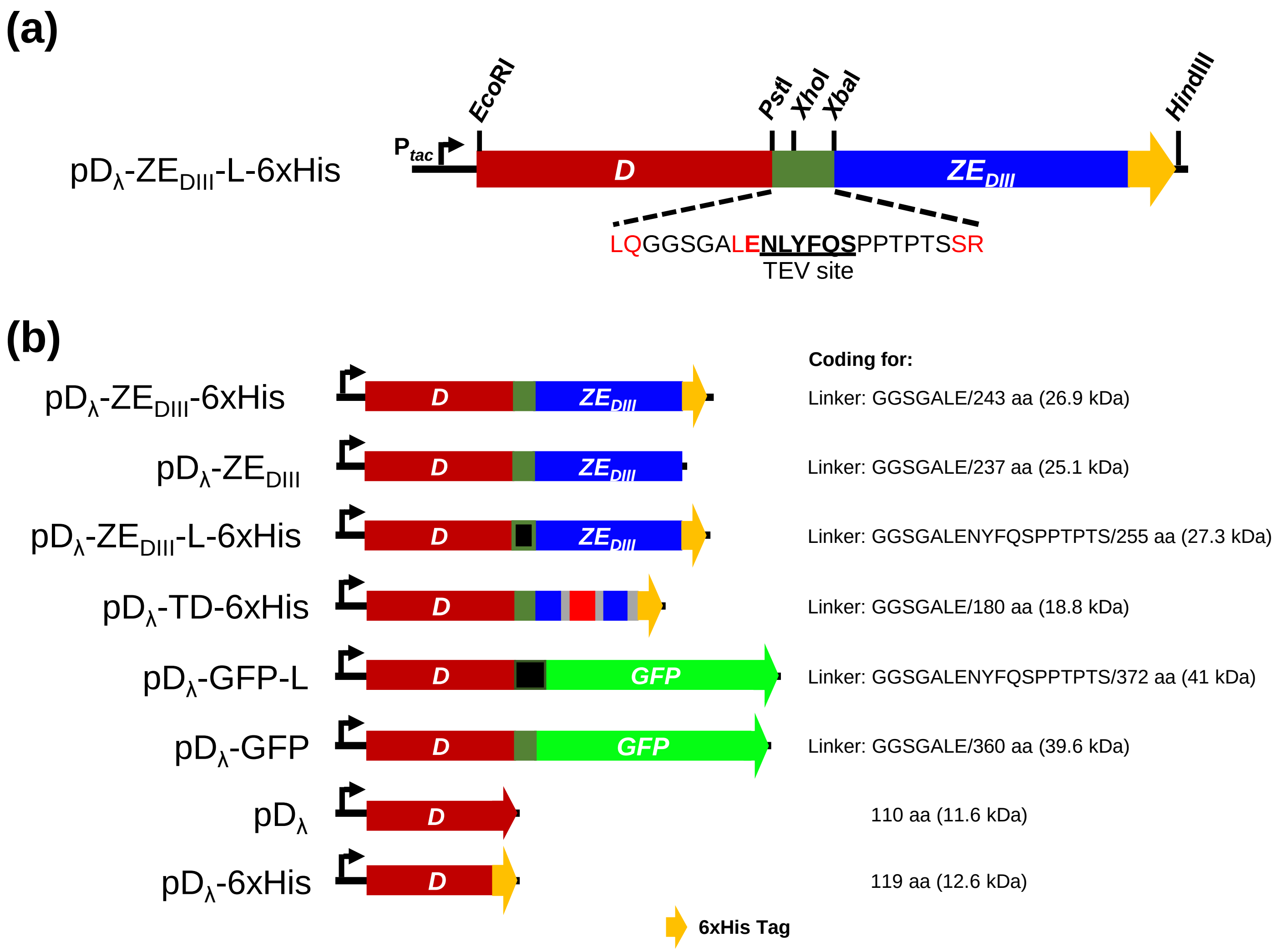

(a)
pDλ-ZEDIII-L-6xHis
Ptac
EcoRI
PstI
XhoI
XbaI
HindIII
D
ZEDIII
LQGGSGALENLYFQSPPTPTSSR
TEV site
(b)
Coding for:
pDλ-ZEDIII-6xHis
Linker: GGSGALE/243 aa (26.9 kDa)
pDλ-ZEDIII
Linker: GGSGALE/237 aa (25.1 kDa)
pDλ-ZEDIII-L-6xHis
Linker: GGSGALENYFQSPPTPTS/255 aa (27.3 kDa)
pDλ-TD-6xHis
Linker: GGSGALE/180 aa (18.8 kDa)
pDλ-GFP-L
GFP
Linker: GGSGALENYFQSPPTPTS/372 aa (41 kDa)
pDλ-GFP
Linker: GGSGALE/360 aa (39.6 kDa)
pDλ
110 aa (11.6 kDa)
pDλ-6xHis
119 aa (12.6 kDa)
6xHis Tag

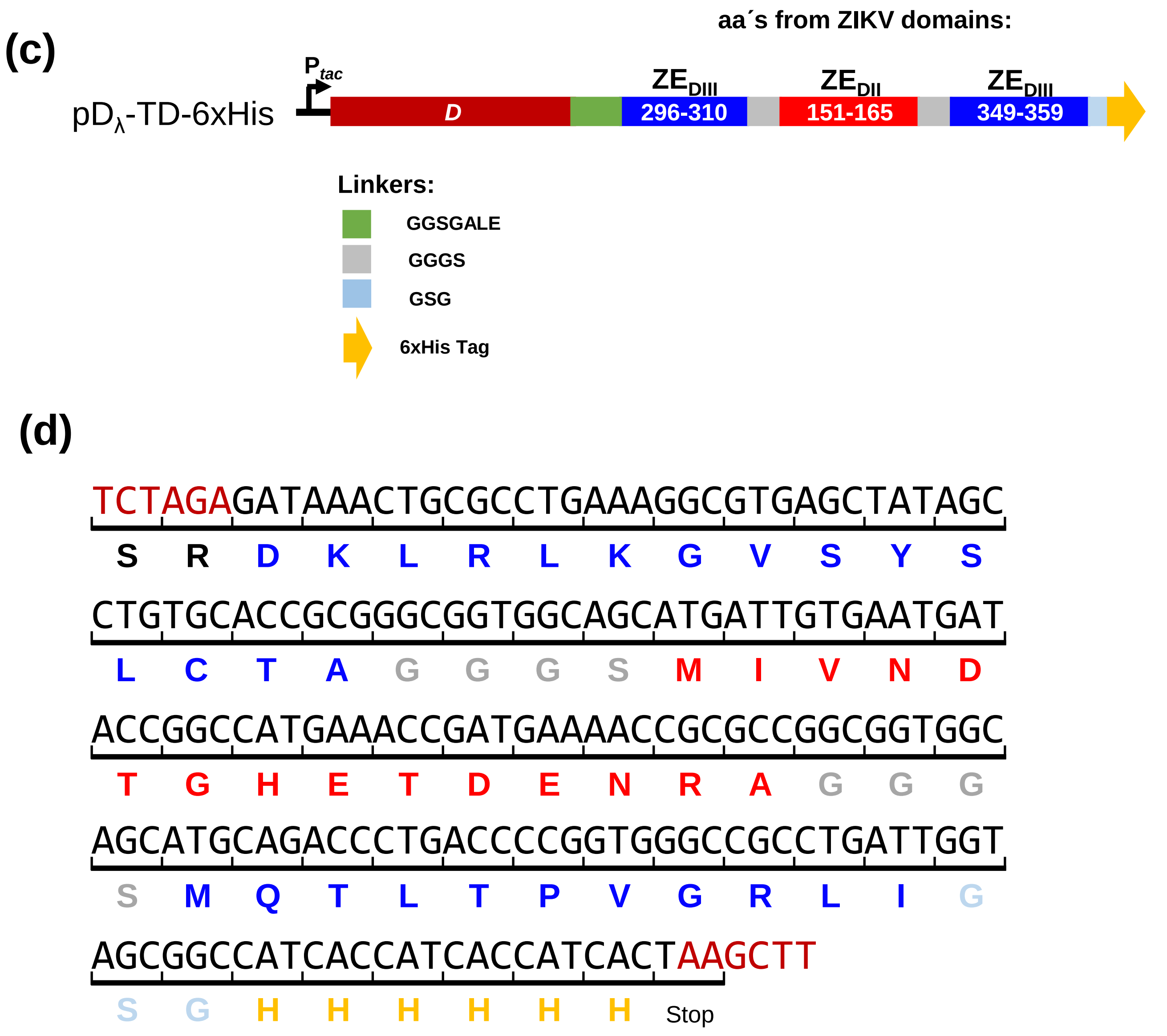

Supplemental Fig. S1

Schematic representation of plasmid constructs for fusion proteins expression. (a) All constructs contain the P_{tac} promoter, represented by an angled arrow; the coding sequence for λ *D* gene is represented by a red rectangle, ZE_{DIII} by a blue rectangle, the linkers between these two by a green square (for short) or a black square (for long), 6xHis by a yellow boxed arrowhead. The amino acids corresponding to the linker are displayed in capital letters. Underlined bold font corresponds to the protease TEV recognition site, and those corresponding to the restriction enzymes are in red font. (b) Based on pD_{λ}-ZE_{DIII}-L-6xHis, different fusions were constructed by substituting the fragment between the *Xba*I-*Hin*dIII sites, the linker between *Pst*I-*Xba*I, or both. The linker used in each construction and the estimated peptide length and molecular weight (kDa) are shown at the right. pD_{λ} and pD_{λ}-6xHis were constructed for use as controls. (c) Schematic representation of the D_{λ}-TD-6xHis fusion. Three different linear epitopes were selected from ZE_{DII} and ZE_{DIII} [Qu, et al. 2020; Antonelli, et al. 2022], their aminoacidic position in E_{ZIKV} is shown, and their nucleotide sequences were optimized for expression in *E. coli*. Short linkers (GGGS) were added to join each adjacent epitope and the 6xHis tag. (d) Complete optimized sequence of TD-6xHis for expression in *E. coli*. Red letters correspond to *Xba*I and *Hin*dIII sites. The nucleotide sequences translated into amino acids are represented by colored letters according to figure c.

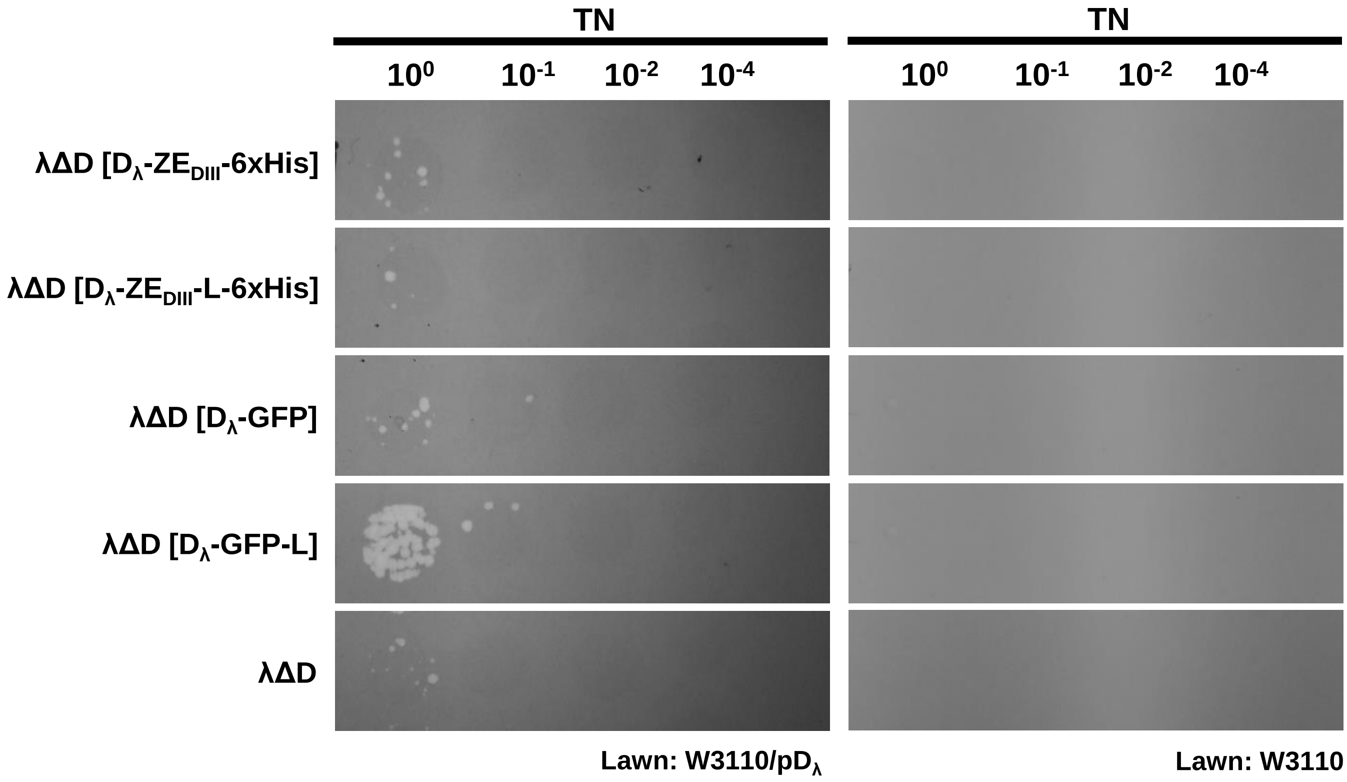

Supplemental Fig. S2

Expression of the wild-type $D_λ$ protein stabilizes the distinct λΔD decorated phages. Production of λΔD phages decorated with different fusion proteins (as indicated at the left) was evaluated by plaque assays using liquid lysates generated by infection of the W3110 strain transformed with the corresponding plasmids. Serial dilutions were prepared with TN (10 mM Tris-HCl pH= 7.8, 100 mM NaCl) buffer, and spotted on different bacterial lawns. Almost all lysates hardly reached a 10^2 PFU/ml titer, and only λΔD [$D_λ$-GFP-L] lysate had a viral titer of 10^3 PFU/ml, when spotted on the W3110/p$D_λ$ lawn (left panel). None generated progeny in absence of $D_λ$ (W3110 lawn).

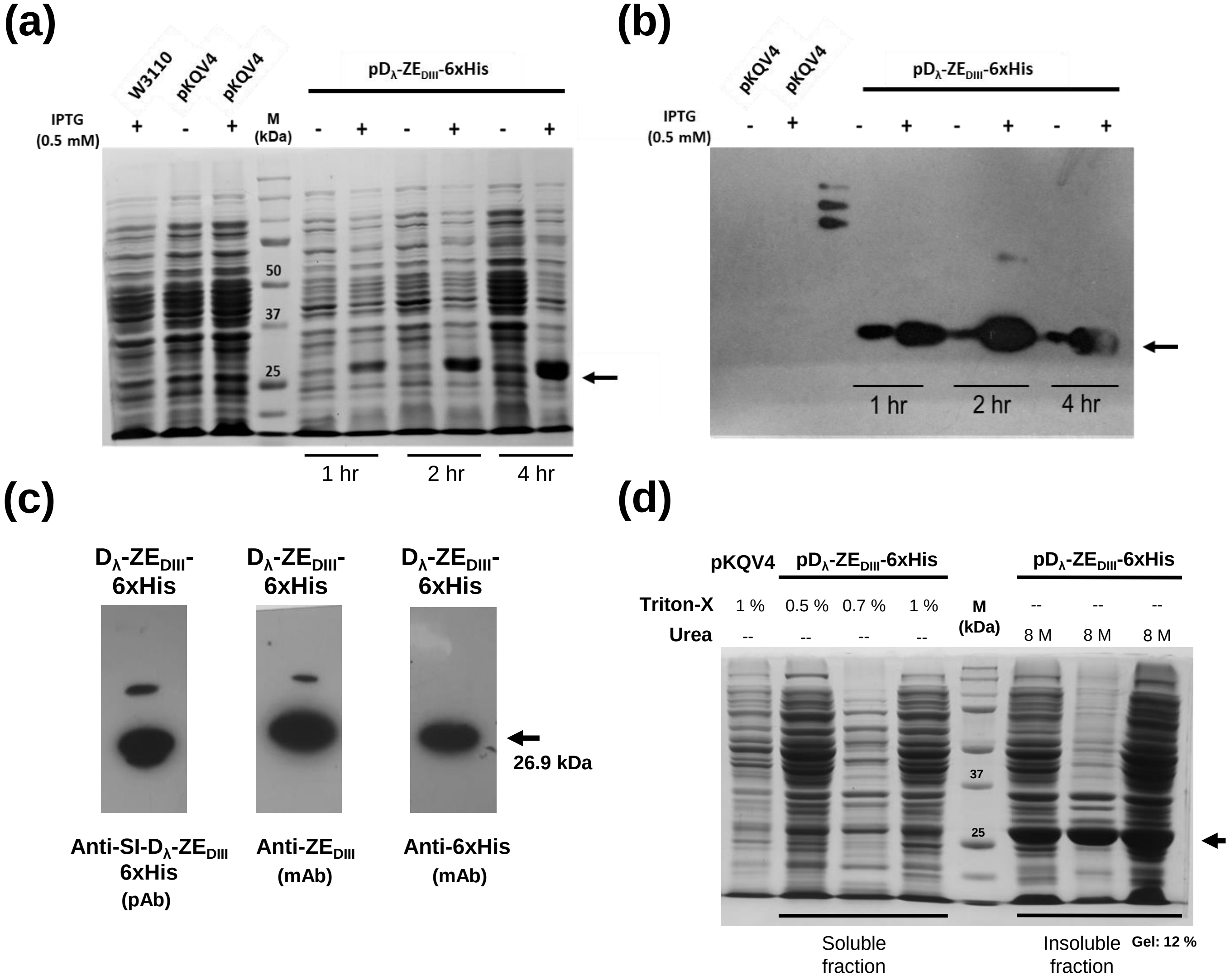

Supplemental Fig. S3

Overexpression of D$_{\lambda}$-ZE$_{DIII}$-6xHis in *E. coli*. (a) SDS-PAGE of D$_{\lambda}$-ZE$_{DIII}$-6xHis protein induction with 0.5 mM of IPTG for 1, 2 and 3 hours in W3110 strain. A discrete band was observed around ~26.9 kDa (black arrow) that increased over time. W3110 and empty vector were used as controls. (b) Immunodetection by western blot of D$_{\lambda}$-ZE$_{DIII}$-6xHis in induced bacterial lysates using an anti-6xHis monoclonal antibody. A signal was observed even in the samples without induction. (c) Western blot immunodetection assays using distinct primary antibodies to detect D$_{\lambda}$-ZE$_{DIII}$-6xHis. In the left panel, the immune serum generated in BALB/c mice was used; commercial anti-ZE$_{DIII}$ and anti-6xHis antibodies were used in the middle and right panel, respectively. Two bands were observed when anti ZE$_{DIII}$ and serum were tested, which could be explained by multimer interactions of D$_{\lambda}$- ZE$_{DIII}$-6xHis. (d) Solubility test of D$_{\lambda}$-ZE$_{DIII}$-6xHis in the W3110 strain. Soluble and insoluble fractions of cell lysates resolved in 12 % SDS-PAGE are shown. The soluble fractions were obtained by cell lysis using a buffer (10 mM Tris-HCl, 100 mM NaCl) with different Triton-X concentrations, as indicated. Insoluble fractions were obtained by treatment of the cell pellets with 8 M urea buffer. The bulk of the D$_{\lambda}$-ZE$_{DIII}$-6xHis protein was observed in the insoluble fraction.

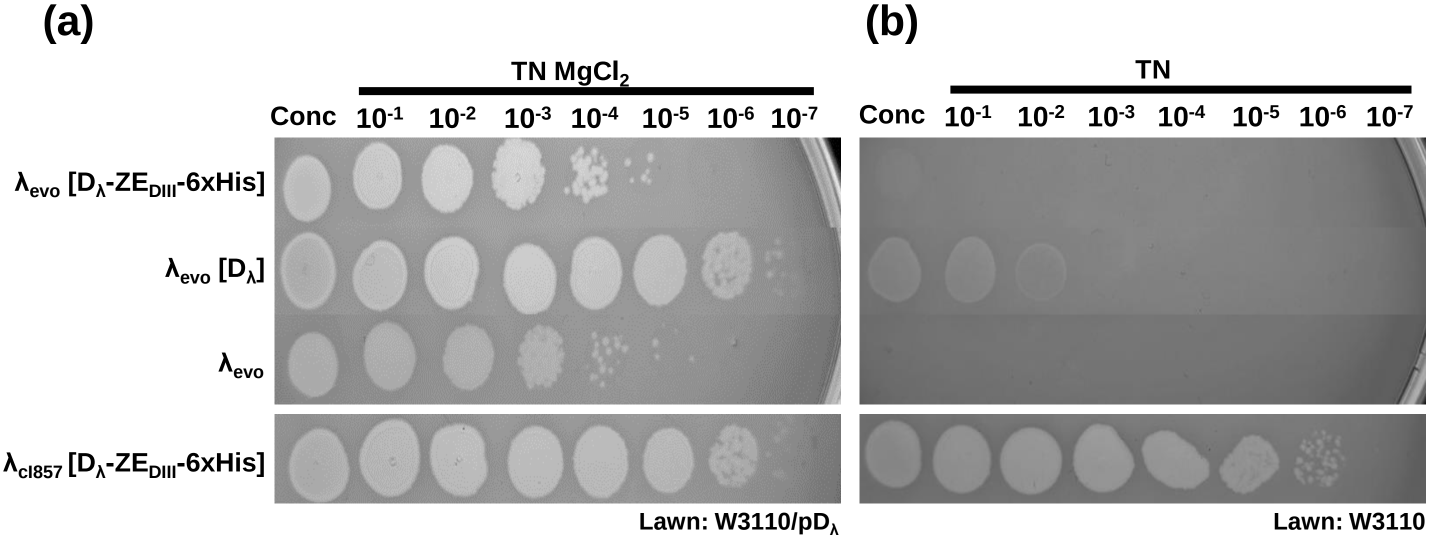

Supplemental Fig. S4

Evaluation of phage viability using $MgCl_2$. (a) Plaque assay of liquid lysates of the distinct λ_{evo} phages decorated with D_{λ}-ZE_{DIII}-6xHis (λ_{evo} [D_{λ}-ZE_{DIII}-6xHis]), D_{λ} (λ_{evo} [D_{λ}]) or none (λ_{evo}). Serial dilutions were prepared with TN (10 mM Tris-HCl pH= 7.8, 100 mM NaCl) buffer containing 10 mM $MgCl_2$, and spotted on W3110/pD_{λ} lawns. Lysate of λ_{cI857} phage in W3110/pD_{λ}-ZE_{DIII}-6xHis was used as control. (b) Samples of (a) were also spotted on a W3110 lawn, where all λ_{evo} phages were unable to produce plaques in absence of D_{λ} complementation.

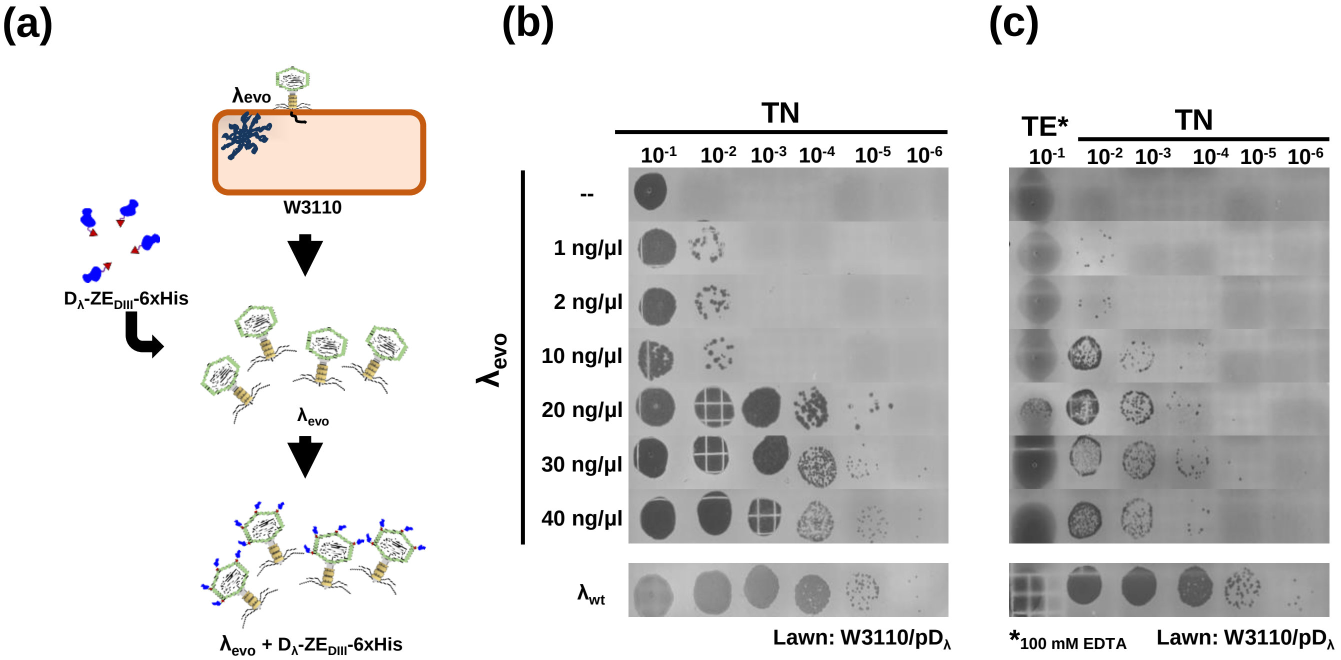

Supplemental Fig. S5

In vitro decoration of λ_{evo} phage. (a) Scheme of the in vitro decoration strategy. The λ_{evo} phage is mixed with purified D_{λ}-ZE_{DIII}-6xHis protein to obtain λ_{evo} + D_{λ}-ZE_{DIII}-6xHis. (b) Plaque assay of λ_{evo} decorated with different D_{λ}-ZE_{DIII}-6xHis protein concentrations. Serial dilutions were prepared on TN (10 mM Tris-HCl pH= 7.8, 100 mM NaCl) buffer, and spotted in W3110/pD_{λ} lawn. 20 ng/µl of purified protein are enough to obtain a viral titer of λ_{evo} + D_{λ}-ZE_{DIII}-6xHis similar to that of λ phage. The serial dilutions were spotted on W3110/pD_{λ} lawns. (c) Effect of EDTA on phage titer. Pre-incubation with 100 mM EDTA (TE buffer) decreased the viral titer of all in vitro-decorated λ_{evo} phage lysates. Even the phage λ_{evo} + D_{λ}-ZE_{DIII}-6xHis decorated with 40 ng/µl of purified protein showed a 100-fold reduced viral titer, compared to λ_{wt} phage.

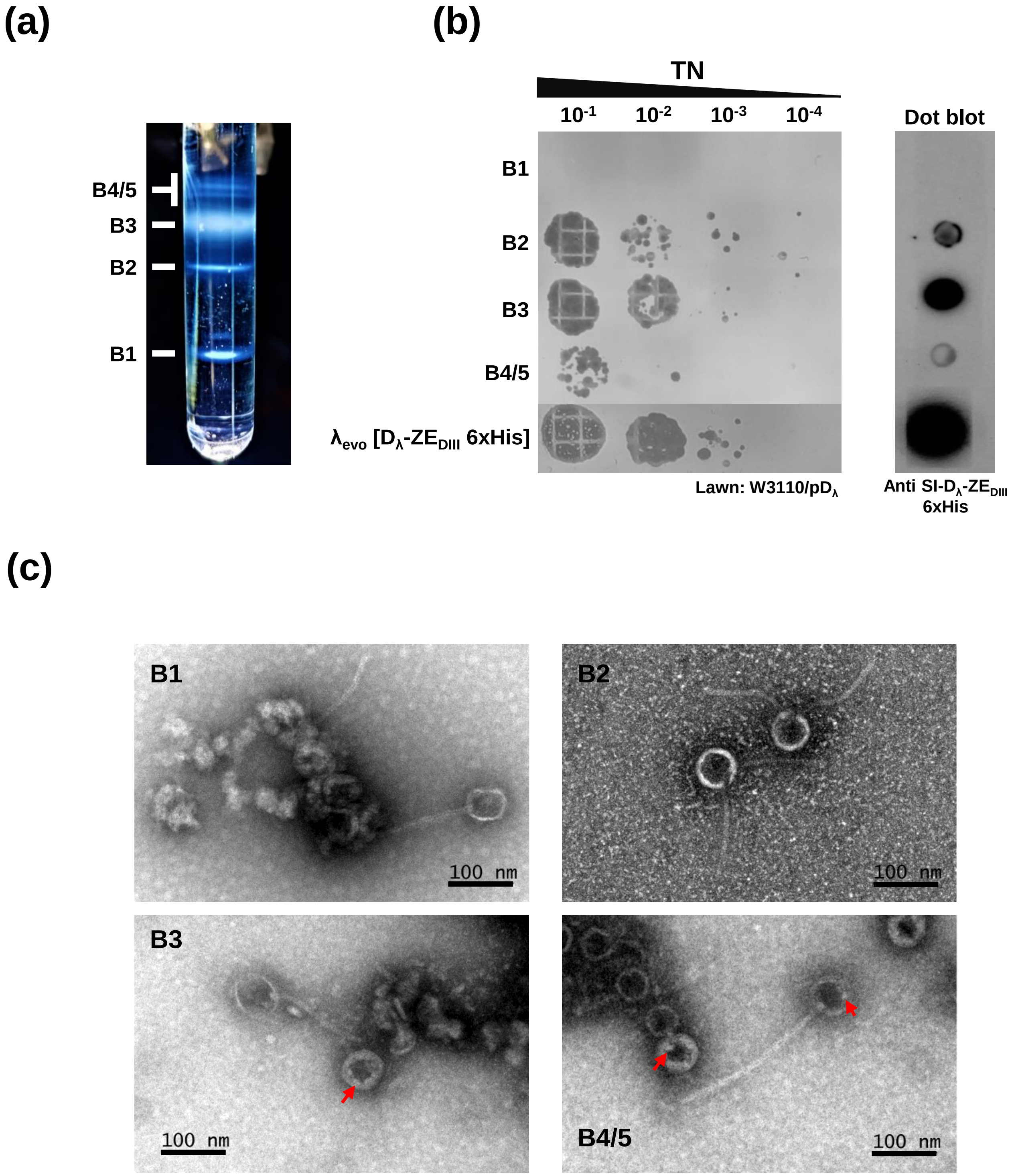
(a)
B4/5
B3
B2
B1
(b)
TN
10^{-1}
10^{-2}
10^{-3}
10^{-4}
Dot blot
B1
B2
B3
B4/5
λ_{evo} [D_{λ}-ZE_{DIII} 6xHis]
Lawn: W3110/pD_{λ}
Anti SI-D_{λ}-ZE_{DIII} 6xHis
(c)
B1
100 nm
B2
100 nm
B3
100 nm
B4/5
100 nm

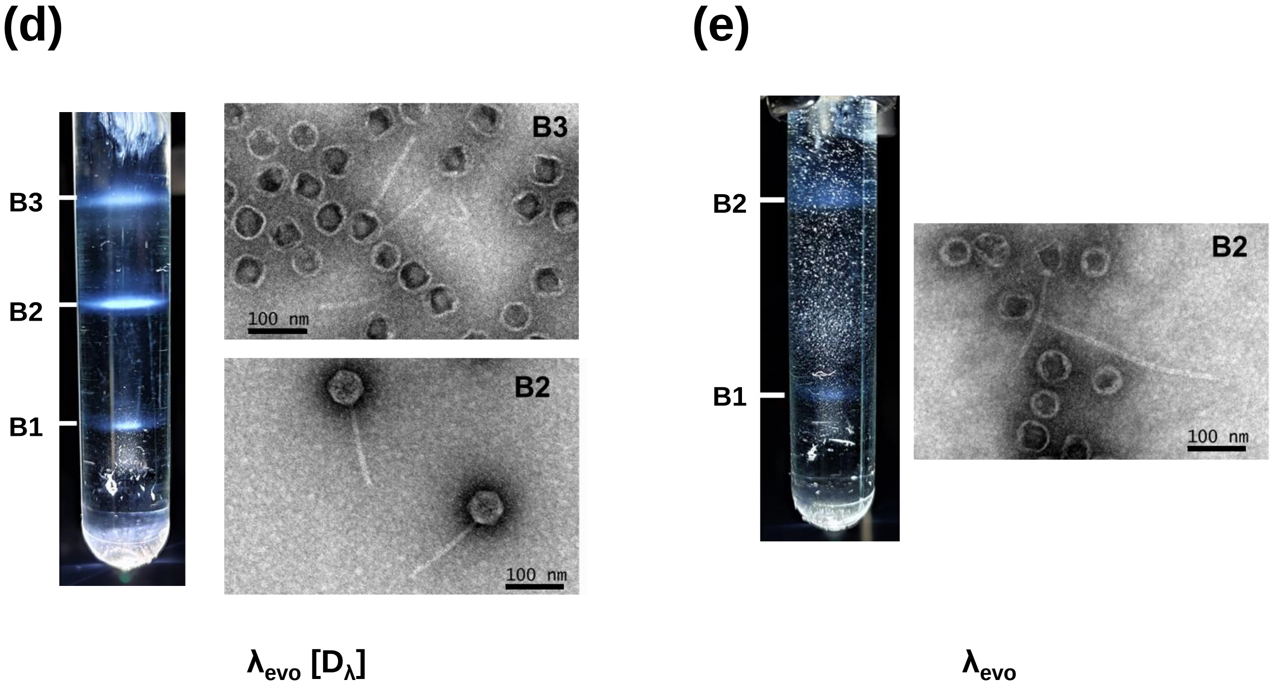

Supplemental Fig. S6

Phage purification by CsCl gradient. (a) CsCl gradient centrifugation of λ_{evo} [D_{λ}-ZE_{DIII}-6xHis] generated 5 discrete bands that were purified (B1, B2, B3; B4 and B5 were recovered as a single sample). (b) Plaque assay of the phages purified from the CsCl-gradient. Phages corresponding to B2 and B3 bands retained the highest viral titer, similar to a liquid lysate sample without CsCl purification. This samples were evaluated by dot blot using SI-D_{λ}-ZE_{DIII}-6xHis as primary antibody. The highest signal observed corresponded to B3, despite it was lower than phages without purification. (c) TEM image of phages recovered from the different CsCl gradient bands. Cellular debris could be observed in the B1 sample; complete phage particles were observed in B2 and B3, and many phages with more than one tail could be observed on B2. Crashed capsids and complete phages could be observed on B3. Small portions of capsids generated a high diffraction of light (red arrows). The B4/B5 sample consisted almost completely in empty pre-capsids. (d) CsCl gradient purification of λ_{evo} [D_{λ}], where 3 phage bands were recovered. Only phages from B2 and B3 could be observed by TEM; B2 showed a λ_{wt}-like population, while B3 mainly consisted in empty pre-capsids. (e) CsCl gradient purification of λ_{evo} without decoration. B2 was the only sample that could be observed by TEM and consisted in empty pre-capsids.

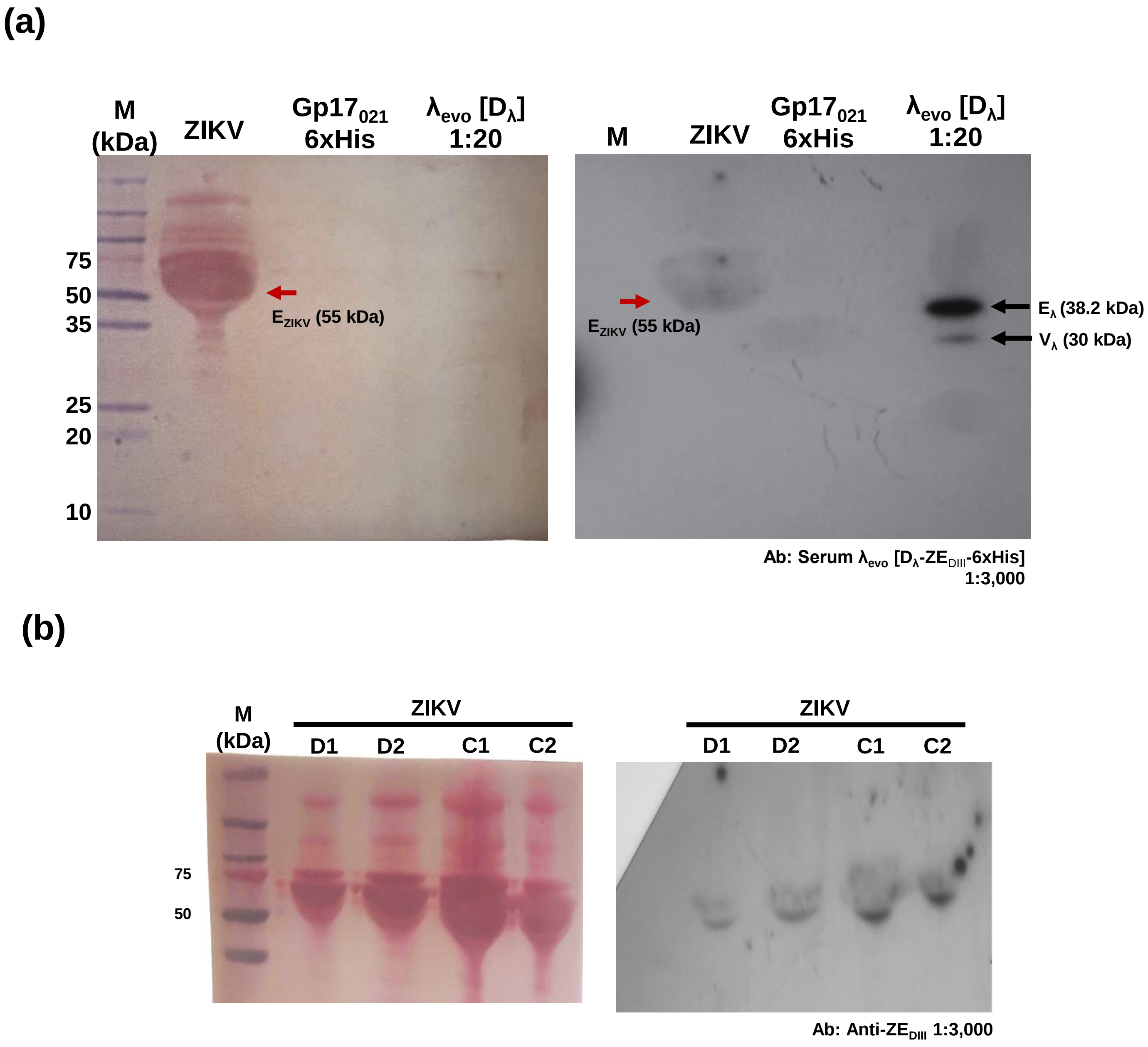

Supplemental Fig. S7

Immunodetection of E_{ZIKV} in ZIKV lysates. (a) Right panel: Immunodetection of different viral lysates and purified proteins with a λ_{evo} [D_λ-ZE_{DIII}-6xHis] serum. Samples are as indicated at the top of the lanes. Immune serum was able to recognize E_{ZIKV}, as shown by a diffuse band; the serum also recognized the E_λ and V_λ proteins from λ_{evo} [D_λ]. Left panel: Ponceau S staining of the proteins transferred to the nitrocellulose membrane. (b) Right panel: Immunodetection of the E_{ZIKV} protein in ZIKV lysates using a monoclonal antibody against ZE_{DIII}. Two different samples of liquid ZIKV lysates (C1 and C2) and its 1:10 dilutions (D1 and D2) were transferred to a nitrocellulose membrane for immunodetection. E_{ZIKV} protein could be observed as a discrete band and a diffuse band above. Left panel: Ponceau S staining of the proteins transferred to the nitrocellulose membrane.

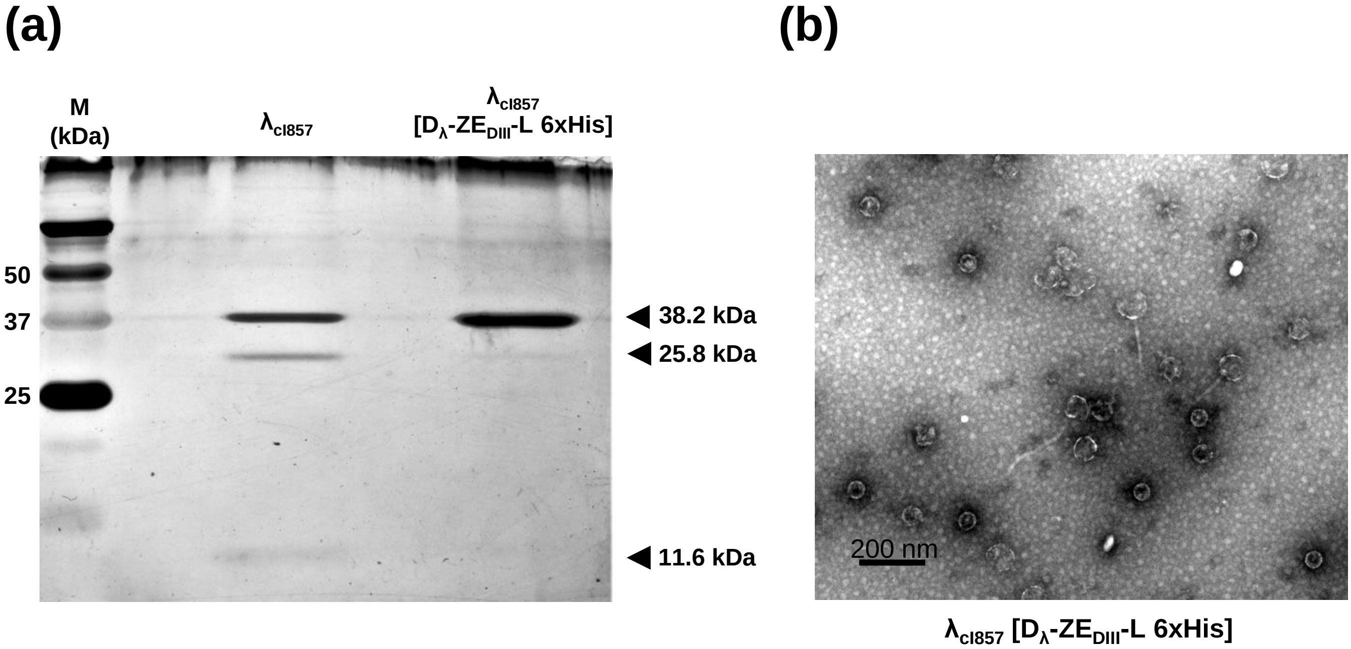

Supplemental Fig. S8

Evaluation of the λ_{cI857} display platform. (a) 16 % SDS-PAGE of the structural proteins of purified phages λ_{cI857} and λ_{cI857} [D_{λ}-ZE_{DIII}-L-6xHis] stained with silver nitrate. No fusion protein was observed in the decorated phage sample. Proteins E_{λ} (38.2 kDa), V_{λ} (25.8 kDa), and D_{λ} (11.4 kDa) were observed in the λ phage sample. Only protein E_{λ} was observed as a discrete band in the λ_{cI857} [D_{λ}-ZE_{DIII}-L-6xHis] sample. (b) TEM image of λ_{cI857} [D_{λ}-ZE_{DIII}-L-6xHis]. Empty precapsids and broken capsids were observed, indicating destabilization of λ_{cI857} phage.

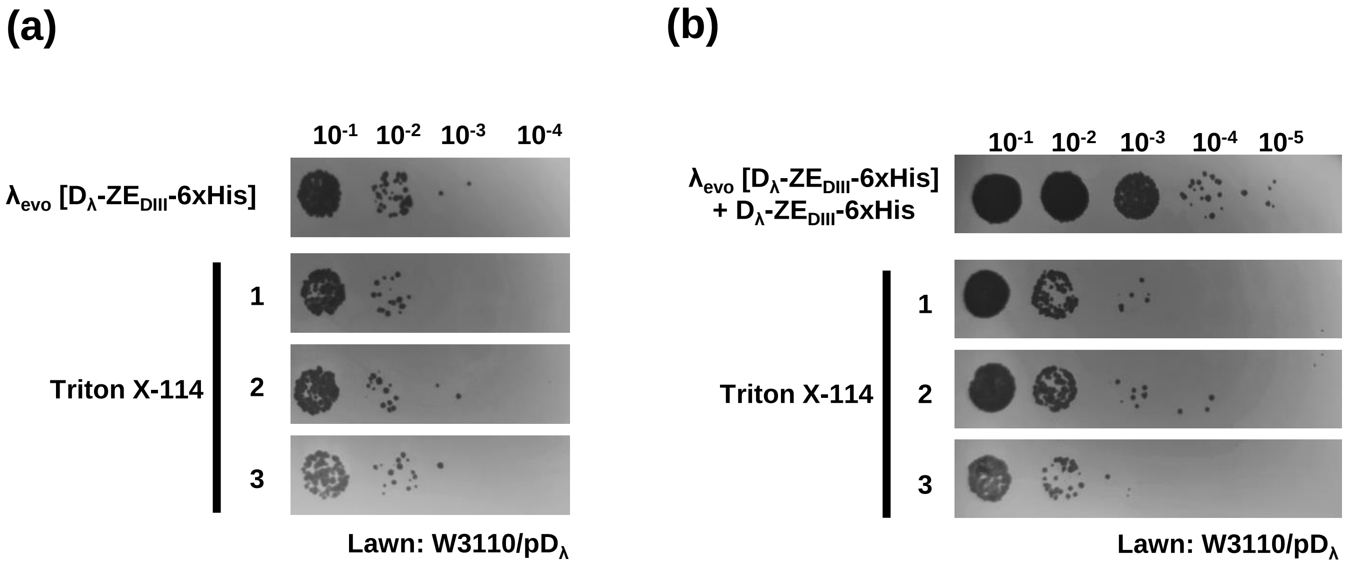

Supplemental Fig. S9
Resistance of λ_{evo} [D_{λ}-ZE_{DIII}-6xHis] and λ_{evo} [D_{λ}-ZE_{DIII}-6xHis] + D_{λ}-ZE_{DIII}-6xHis to Triton X-114 treatment. (a) Plaque assay of in vivo decorated λ_{evo} [D_{λ}-ZE_{DIII}-6xHis] before and after 3 treatments with Triton X-114. The viral titer was not affected after treatment. (b) Plaque assay of in vivo/in vitro decorated λ_{evo} [D_{λ}-ZE_{DIII}-6xHis] + D_{λ}-ZE_{DIII}-6xHis before and after 3 treatments with Triton X-114. Viral titer was reduced 10-fold after the first Triton X-114 treatment and remained similar to the titer observed in the in vivo decoration experiments shown in (A).

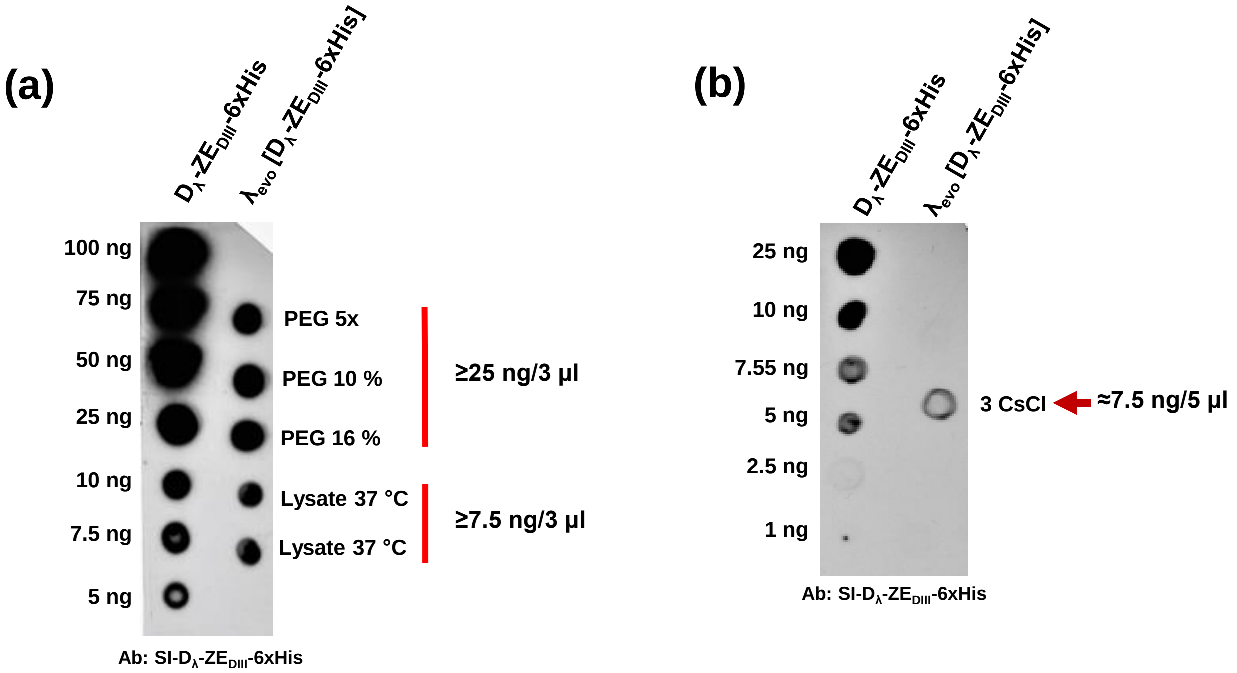

Supplemental Fig. S10

Estimation of the amount of D_λ-ZE_{DIII}-6xHis protein in different samples of λ_{evo} [D_λ-ZE_{DIII}-6xHis] phage. (a) Semi-quantitative assay based on dot blot immunodetection, using purified D_λ-ZE_{DIII}-6xHis as reference to estimate the amount of D_λ-ZE_{DIII}-6xHis displayed in λ_{evo} [D_λ-ZE_{DIII}-6xHis]. Lysates with different PEG concentration generated a signal equivalent to ~8 ng/µl of fusion protein, while lysates without PEG emitted a signal equivalent to ~2.5 ng/µl. (b) A similar assay using λ_{evo} [D_λ-ZE_{DIII}-6xHis] phage purified by CsCl gradient. The most concentrated band (3 CsCl) was recovered and evaluated, generating a signal equivalent to ~1.3 ng/µl of protein.

References

- Antonelli ACB, Almeida VP, Castro FOF de, Silva JM, Pfrimer IAH, Cunha-Neto E, Maranhão AQ, Brígido MM, Resende RO, Bocca AL, Fonseca SG (2022) In silico construction of a multiepitope Zika virus vaccine using immunoinformatics tools. Sci Rep 12:53. https://doi.org/10.1038/s41598-021-03990-6
- Baba T, Ara T, Hasegawa M, Takai Y, Okumura Y, Baba M, Datsenko KA, Tomita M, Wanner BL, Mori H (2006) Construction of *Escherichia coli* K-12 in-frame, single-gene knockout mutants: the Keio collection. Mol Syst Biol 2:2006.0008-2006.0008. https://doi.org/10.1038/msb4100050
- Bachmann BJ (1972) Pedigrees of some mutant strains of *Escherichia coli* K-12. Bacteriol Rev 36:525–57. https://doi.org/10.1128/br.36.4.525-557.1972
- Datsenko KA, Wanner BL (2000) One-step inactivation of chromosomal genes in *Escherichia coli* K-12 using PCR products. Proc Natl Acad Sci United States Am 97:6640–5. https://doi.org/10.1073/pnas.120163297
- Deatherage CL, Hadziselimovic A, Sanders CR (2012) Purification and characterization of the human γ-secretase activating protein. Biochemistry 51:5153–5159. https://doi.org/10.1021/bi300605u
- Hanahan D (1983) Studies on transformation of *Escherichia coli* with plasmids. J Mol Biol 166:557–580. https://doi.org/10.1016/s0022-2836(83)80284-8
- Qu P, Zhang C, Li M, Ma W, Xiong P, Liu Q, Zou G, Lavillette D, Yin F, Jin X, Huang Z (2019) A new class of broadly neutralizing antibodies that target the glycan loop of Zika virus envelope protein. Cell Discov 6:5. https://doi.org/10.1038/s41421-019-0140-8
- Strauch MA, Perego M, Burbulys D, Hoch JA (1989) The transition state transcription regulator AbrB of *Bacillus subtilis* is autoregulated during vegetative growth. Mol Microbiol 3:1203–1209. https://doi.org/10.1111/j.1365-2958.1989.tb00270.x
- Thermo Fisher Scientific (2024) CloneJET PCR cloning kit
